# Supplementary material for: Association between exercise habits and stroke, heart failure, and mortality in Korean patients with incident atrial fibrillation: A nationwide population-based cohort study
Source: PLoS Med. 2021 Jun 8;18(6):e1003659. doi: 10.1371/journal.pmed.1003659 (PMC8219164; doi:10.1371/journal.pmed.1003659)
Supplement: S1 STROBE Checklist — (DOC) [file pmed.1003659.s001.doc]

STROBE Statement—Checklist of items that should be included in reports of ***cohort studies***

|  | Item No | Recommendation |
| --- | --- | --- |
| **Title and abstract** | 1 | 1. Indicate the study’s design with a commonly used term in the title or the abstract   ***The study design is indicate by the term “a cohort study” in the title, “a nationwide population-based cohort study” in the Abstract*** |
|  |  | 1. Provide in the abstract an informative and balanced summary of what was done and what was found   ***We believe we have provided an informative and balanced summary of what was done and what was found in the abstract.*** |
| Introduction | | |
| Background/rationale | 2 | Explain the scientific background and rationale for the investigation being reported  ***We have explained the scientific background and rationale for the investigations being reported in the introduction paragraphs 1 and 2.*** |
| Objectives | 3 | State specific objectives, including any prespecified hypotheses  ***The specific objectives and hypotheses are stated in the introduction paragraph 3.*** |
| Methods | | |
| Study design | 4 | Present key elements of study design early in the paper  ***The key elements of study design are presented in the methods section in paragraphs 2-4 and Fig 1.*** |
| Setting | 5 | Describe the setting, locations, and relevant dates, including periods of recruitment, exposure, follow-up, and data collection  ***The setting, locations, periods of recruitment, dates of recruitment; details on follow-up and data collection relevant to this manuscript are given in the methods section in paragraphs 2-6.*** |
| Participants | 6 | 1. *Cross-sectional study—Give the eligibility criteria, and the sources and methods of selection of participants*   ***The eligibility criteria for this study and the sources and methods of selection of participants is given in the methods section in paragraphs 1-2 and Fig 1.*** |
|  |  |  |
| Variables | 7 | Clearly define all outcomes, exposures, predictors, potential confounders, and effect modifiers. Give diagnostic criteria, if applicable  ***All outcomes, exposures are defined in the methods section in paragraphs 2.*** |
| Data sources/ measurement | 8* | For each variable of interest, give sources of data and details of methods of assessment (measurement). Describe comparability of assessment methods if there is more than one group  ***Sources of data and details of methods of assessment are given in the methods section in paragraphs 1-6.*** |
| Bias | 9 | Describe any efforts to address potential sources of bias  ***Any efforts to address potential sources of bias are described in the methods section in paragraphs 7-10.*** |
| Study size | 10 | Explain how the study size was arrived at  ***An explanation of the study size is given in the methods section in paragraph 2.*** |
| Quantitative variables | 11 | Explain how quantitative variables were handled in the analyses. If applicable, describe which groupings were chosen and why  ***An explanation of how quantitative variables were handled in the analyses is given in the methods section in paragraph 7.*** |
| Statistical methods | 12 | 1. Describe all statistical methods, including those used to control for confounding   ***All statistical methods are given in the methods section in paragraph 7-10.***  ***The study authorship includes an experienced medical statistician.*** |
|  |  | 1. Describe any methods used to examine subgroups and interactions   ***Any methods used to examine subgroups and interactions are given in the methods section in paragraphs 8-10.*** |
|  |  | 1. Explain how missing data were addressed   ***Study subjects with missing data were excluded in the analysis as described in Fig 1.*** |
|  |  | 1. *Cross-sectional study—If applicable, describe analytical methods taking account of sampling strategy*   ***Not applicable.*** |
|  |  | 1. Describe any sensitivity analyses   ***Sensitivity analysis was performed and described in paragraph 10.*** |

Continued on next page

| Results | | |
| --- | --- | --- |
| Participants | 13* | 1. Report numbers of individuals at each stage of study—eg numbers potentially eligible, examined for eligibility, confirmed eligible, included in the study, completing follow-up, and analysed   ***The numbers of individuals at each stage of the study are reported in the Results section in paragraph 1-2 and Fig 1.*** |
|  |  | 1. Give reasons for non-participation at each stage   ***The numbers and reasons for subjects of non-participation are reported in Fig 1. and eTable2.*** |
|  |  | 1. Consider use of a flow diagram   ***A flow diagram is described in Fig 1.*** |
| Descriptive data | 14* | 1. Give characteristics of study participants (eg demographic, clinical, social) and information on exposures and potential confounders   ***Characteristics of study participants are given in Table 1 and reported in paragraph 1-2 of the Results section.*** |
|  |  | 1. Indicate number of participants with missing data for each variable of interest   ***The number of participants with missing data is reported in the Fig 1 and eTable2.*** |
|  |  | (c) Summarise follow-up time (eg, average and total amount)  ***The follow-up times of each outcome are reported in the Results section in paragraph 3*** |
| Outcome data | 15* | *Cross-sectional study—Report numbers of outcome events or summary measures*  ***Numbers of outcome events are given in the Results section paragraphs 4-11, Table 2 and in all the Tables in the online supplement.*** |
| Main results | 16 | 1. Give unadjusted estimates and, if applicable, confounder-adjusted estimates and their precision (eg, 95% confidence interval). Make clear which confounders were adjusted for and why they were included   ***Full results are given in Table 2 and in all the Tables in the online supplement. Confounders adjusted for are clearly described in Method section.*** |
|  |  | 1. Report category boundaries when continuous variables were categorized   ***Category boundaries for continuous variables are reported in Table 2.*** |
|  |  | 1. If relevant, consider translating estimates of relative risk into absolute risk for a meaningful time period   ***Absolute risk, absolute risk reduction, and numbers needed to treat values are described in Table 2 and Results section.*** |
| Other analyses | 17 | Report other analyses done—eg analyses of subgroups and interactions, and sensitivity analyses  ***Subgroup analyses and additional analyses in response to peer review are reported in Results section in paragraphs 9-10.*** |
| Discussion | | |
| Key results | 18 | Summarise key results with reference to study objectives  ***Key results are summarised with reference to study objectives in the first paragraph of the Discussion section.*** |
| Limitations | 19 | Discuss limitations of the study, taking into account sources of potential bias or imprecision. Discuss both direction and magnitude of any potential bias  ***Limitations of the study are discussed in the Discussion section in paragraph 8.*** |
| Interpretation | 20 | Give a cautious overall interpretation of results considering objectives, limitations, multiplicity of analyses, results from similar studies, and other relevant evidence  ***An overall interpretation of results is given in the Discussion section in paragraphs 2-7.*** |
| Generalisability | 21 | Discuss the generalisability (external validity) of the study results  ***Generalisability is discussed in the Discussion section in paragraph 8.*** |
| Other information | | |
| Funding | 22 | Give the source of funding and the role of the funders for the present study and, if applicable, for the original study on which the present article is based  ***The sources of funding and the role of funding for the study is reported separately during manuscript submission as per PLOS Medicine guidelines.*** |

*Give information separately for cases and controls in case-control studies and, if applicable, for exposed and unexposed groups in cohort and cross-sectional studies.

**Note:** An Explanation and Elaboration article discusses each checklist item and gives methodological background and published examples of transparent reporting. The STROBE checklist is best used in conjunction with this article (freely available on the Web sites of PLoS Medicine at http://www.plosmedicine.org/, Annals of Internal Medicine at http://www.annals.org/, and Epidemiology at http://www.epidem.com/). Information on the STROBE Initiative is available at www.strobe-statement.org.
