# Supplementary material for: Association between exercise habits and stroke, heart failure, and mortality in Korean patients with incident atrial fibrillation: A nationwide population-based cohort study
Source: PLoS Med. 2021 Jun 8;18(6):e1003659. doi: 10.1371/journal.pmed.1003659 (PMC8219164; doi:10.1371/journal.pmed.1003659)
Supplement: S1 Fig — CI, confidence interval. Model 1 adjusted for age and sex. Model 2 adjusted for body mass index (BMI), smoking, heavy drinking, and low income in addition to the variables in model 1. Model 3 adjusted for diabetes mellitus, hypertension, dyslipidemia, and previous myocardial infarction (MI) in addition to the variables in model 2. Model 4 adjusted for peripheral artery disease (PAD), chronic obstructive pulmonary disease (COPD), cancer, and chronic kidney disease (CKD) in addition to the variables in model 3. Model 5 adjusted for CHA2DS2-VASc score in addition to the variables in model 4. Model 6 adjusted for use of oral anticoagulation (OAC), use of antiplatelet agent, and use of statin in addition to the variables in model 5. p-Values were evaluated by the likelihood ratio test. The dots denote hazard ratios, and the whiskers denote 95% confidence intervals computed by multivariable Cox proportional hazards models. (DOCX) [file pmed.1003659.s002.docx]

**S1 Fig.** Hazard ratios with 95% confidence intervals for ischemic stroke, heart failure, and all-cause death according to the change of exercise status calculated from the various multivariable-adjusted Cox proportional hazard models.

**
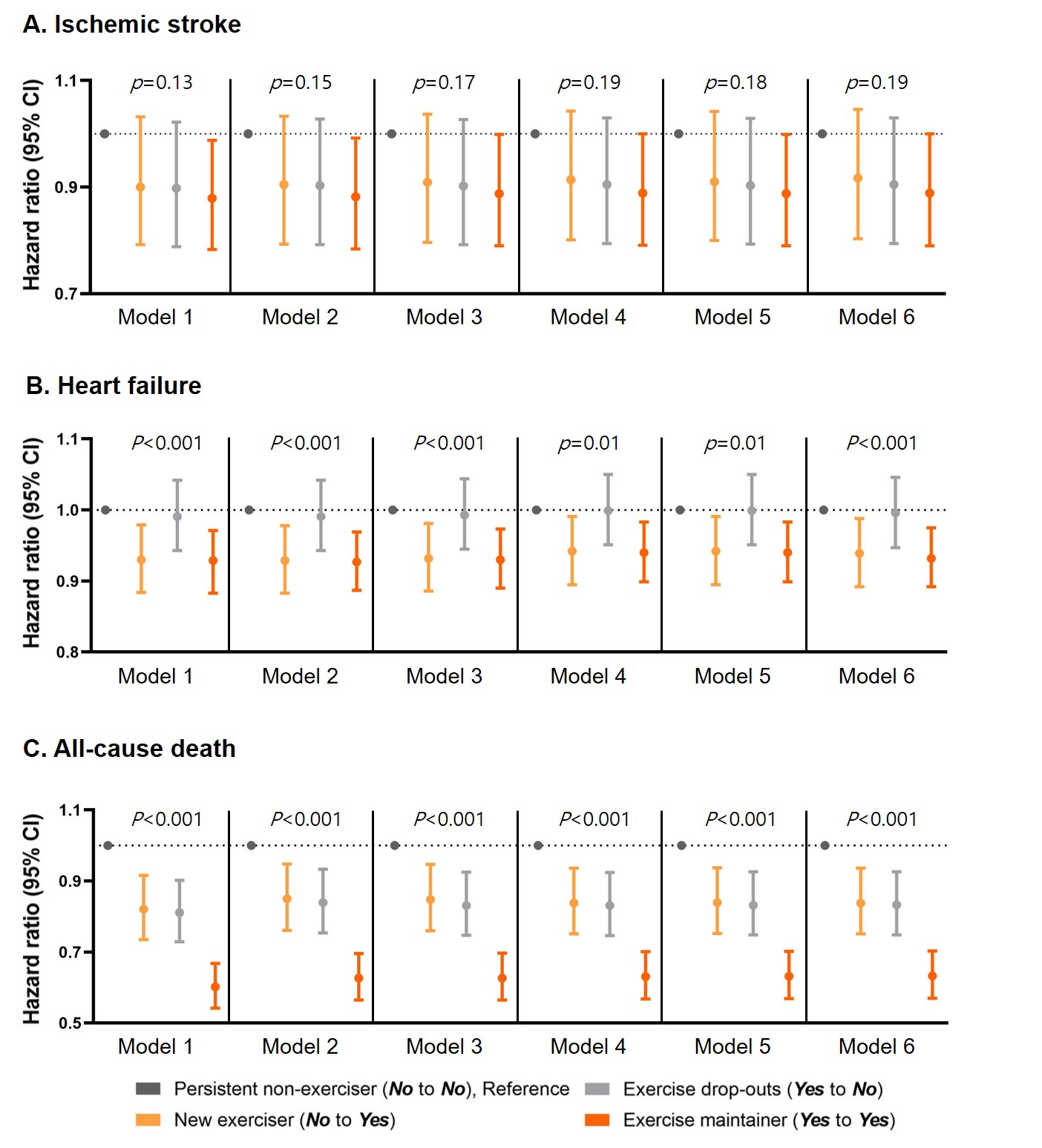
**

Abbreviation: CI, confidence interval.

Model 1 adjusted for age and sex.

Model 2 adjusted body mass index (BMI), smoking, heavy drinking, and low income in addition to Model 1.

Model 3 adjusted diabetes mellitus, hypertension, dyslipidemia, and previous myocardial infarction (MI) in addition to Model 2.

Model 4 adjusted peripheral artery disease (PAD), chronic obstructive pulmonary disease (COPD), cancer, and chronic kidney disease (CKD) in addition to Model 3.

Model 5 adjusted CHA_2_DS_2_-VASc score in addition to Model 4.

Model 6 adjusted the use of oral anticoagulation (OAC), use of antiplatelet agents in addition to Model 5.

*P* values were evaluated by the likelihood ratio test. The dots denote hazard ratios, and the whiskers denote 95% confidence intervals computed by multivariable Cox proportional hazards models.
