# Supplementary material for: Association between exercise habits and stroke, heart failure, and mortality in Korean patients with incident atrial fibrillation: A nationwide population-based cohort study
Source: PLoS Med. 2021 Jun 8;18(6):e1003659. doi: 10.1371/journal.pmed.1003659 (PMC8219164; doi:10.1371/journal.pmed.1003659)
Supplement: S1 Table — CKD, chronic kidney disease; COPD, chronic obstructive pulmonary disease; MI, myocardial infarction; N/A, not applicable; PAD, peripheral artery disease. *Combination: 1 = ICD-10-CM code and medication; 2 = number of diagnosis; and 3 = diagnostic tests or treatment. (DOCX) [file pmed.1003659.s003.docx]

**S1 Table.** Definition of covariates and outcomes.

| **Diagnosis** | **ICD-10-CM code and medication** | **Number of diagnosis** | **Diagnosis test or treatment** | **Combination*** |
| --- | --- | --- | --- | --- |
| **Inclusion/Exclusion** |  |  |  |  |
| Atrial fibrillation | I48.0-48.4, I48.9 | Admission ≥ 1 or outpatient department ≥ 2 | N/A | 1+2 |
| Valvular atrial fibrillation | I05.0, I05.2, I05.9, Z95.2-Z95.4 | Admission ≥ 1 or outpatient department ≥ 1 | N/A | 1+2 |
| **Outcome** |  |  |  |  |
| Ischemic stroke | I63, I64 | Admission ≥ 1 or outpatient department ≥ 1 | Brain imaging (CT or MRI) ≥1 or  Concomitant imaging studies of the brain or related death | 1+2+3 |
| Heart failure | I50 | Admission ≥ 1 or outpatient department ≥ 1 | N/A | 1+2 |
| **Comorbidities based on the 2nd health examination (Health examination after AF diagnosis)** | | |  |  |
| **Comorbidities** |  |  |  |  |
| Hypertension | I10-I13, I15; and minimum 1 prescription of anti-hypertensive drug (thiazide, loop diuretics, aldosterone antagonist, alpha-/beta-blocker, calcium-channel blocker, angiotensin-converting enzyme inhibitor, or angiotensin II receptor blocker) | Admission ≥ 1 or outpatient department ≥ 2 | Systolic/diastolic blood pressure ≥ 140/90 mmHg | 1+2 or 3 |
| Diabetes mellitus | E11-E14; and minimum 1 prescription of anti-diabetic drugs (sulfonylureas, metformin, meglitinides, thiazolidinediones, dipeptidyl peptidase-4 inhibitors, α-glucosidase inhibitors, or insulin) | Admission ≥ 1 or outpatient department ≥ 2 | Fasting glucose level ≥ 126 mg/dL | 1+2 or 3 |
| Dyslipidemia | E78 | Admission ≥ 1 or outpatient department ≥ 1 | Total cholesterol ≥ 240 mg/dL | 1+2 or 3 |
| Previous MI | I21, I22 | Admission ≥ 1 or outpatient department ≥ 1 | N/A | 1+2 |
| PAD | I70, I73 | Admission ≥ 1 or outpatient department ≥ 1 | N/A | 1+2 |
| COPD | J41-44 | Admission ≥ 1 | N/A | 1+2 |
| CKD | N/A | N/A | eGFR<60ml/min/1.73m^2^ | 3 |
| Cancer | C00-97 and RID code (V193) | Admission ≥ 1 or outpatient department ≥ 1 | N/A | 1+2 |
| **Definitions of life style behavior based on the response of 2nd health examination questionnaire (Health examination after AF diagnosis)** | | | | |
| **Alcohol consumption** |  |  |  |  |
| Mild to moderate drinker | Alcohol consumption > 0g to < 30g per day | |  |  |
| Heavy drinker | Alcohol consumption ≥ 30g per day | |  |  |
| **Smoking** |  |  |  |  |
| Ex-smoker | Ex-smoker at the 1st examination and sustaining non-smoking till the 2nd examination | | | |
| Current smoker | Current smoker at the 2nd examination regardless of the smoking status at the 1st examination. | | | |
| **Information of income** | | | | |
| Low income | Income belongs to lower 20% among the entire Korean population and supported by the Medical Aid program | | |  |

Abbreviation: N/A, not applicable; MI, myocardial infarction; PAD, peripheral artery disease; COPD, chronic obstructive pulmonary disease; CKD, chronic kidney disease

* Combination: 1= ICD-10-CM code and medication; 2 = Number of diagnosis; and 3 = Diagnostic tests or treatment
