# Supplementary material for: Association between exercise habits and stroke, heart failure, and mortality in Korean patients with incident atrial fibrillation: A nationwide population-based cohort study
Source: PLoS Med. 2021 Jun 8;18(6):e1003659. doi: 10.1371/journal.pmed.1003659 (PMC8219164; doi:10.1371/journal.pmed.1003659)
Supplement: S2 Table — Data are presented as mean ± standard deviation or number (percentage). Percentages may not total 100 because of rounding. AF, atrial fibrillation; COPD, chronic obstructive pulmonary disease; DOAC, direct oral anticoagulant; HF, heart failure; MI, myocardial infarction; PAD, peripheral artery disease. *Low income denotes income in the lowest 20% among the entire Korean population; individuals with low income are supported by the medical aid program. (DOCX) [file pmed.1003659.s004.docx]

**S2 Table.** Baseline characteristics of newly diagnosed atrial fibrillation patients according to the number of health examinations before and after their atrial fibrillation diagnosis.

|  | Exam 0 | Exam 1 | Exam 2 | p-value |
| --- | --- | --- | --- | --- |
| No. of participants (%) | 225915 | 230567 | 66692 |  |
| Age | 69.04 ± 18.08 | 66.99 ± 12.54 | 59.52 ± 12.38 | < 0.001 |
| Sex |  |  |  | < 0.001 |
| Male | 107632 (47.88) | 130002 (56.38) | 42410 (63.59) |  |
| Female | 117179 (52.12) | 100565 (43.62) | 24282 (36.41) |  |
| Diabetes mellitus | 63499 (28.11) | 56669 (24.58) | 10499 (15.74) | < 0.001 |
| Hypertension | 169424 (74.99) | 177900 (77.16) | 39266 (58.88) | < 0.001 |
| Dyslipidemia | 83578 (37.00) | 102474 (44.44) | 2088 (3.13) | < 0.001 |
| Previous MI | 19780 (8.76) | 15998 (6.94) | 2221 (3.33) | < 0.001 |
| PAD | 44391 (19.65) | 52917 (22.95) | 12053 (18.07) | < 0.001 |
| COPD | 63115 (27.94) | 57812 (25.07) | 10640 (15.95) | < 0.001 |
| Cancer | 25609 (11.34) | 23008 (9.98) | 4197 (6.29) | < 0.001 |
| CHA_2_DS_2_-VASc score | 3.90 ± 2.12 | 3.51 ± 1.89 | 1.91 ± 1.28 | < 0.001 |
| Medications |  |  |  |  |
| Warfarin | 12958 (5.74) | 18494 (8.02) | 9016 (13.52) | < 0.001 |
| DOAC | 5446 (2.41) | 8782 (3.81) | 3374 (5.06) | < 0.001 |
| ASP | 27829 (12.32) | 39171 (16.99) | 13263 (19.89) | < 0.001 |
| P2Y_12_ inhibitor | 12963 (5.74) | 16558 (7.18) | 2834 (4.25) | < 0.001 |
| Statin | 25690 (11.37) | 39137 (16.97) | 9865 (14.79) | < 0.001 |
| Low income * | 70312 (31.12) | 43510 (18.87) | 10817 (16.22) | < 0.001 |
| **Exclusion Criteria** |  |  |  |  |
| Valvular AF | 1574 (0.70) | 2327 (1.01) | 0 (0.00) | < 0.001 |
| Age < 20 years | 4575 (2.04) | 68 (0.03) | 0 (0.00) | < 0.001 |
| Previous history of stroke | 59345 (26.27) | 53949 (23.40) | 0 (0.00) | < 0.001 |
| Previous history of HF | 78493 (34.74) | 82916 (35.96) | 0 (0.00) | < 0.001 |

Data are presented as means ± standard deviation or No. (Percentages).

Percentages may not total 100 because of rounding.

Abbreviation: MI, myocardial infarction; PAD, peripheral artery disease; COPD, chronic obstructive pulmonary disease; DOAC, direct oral anticoagulant; AF, atrial fibrillation; HF, heart failure.

* Low income denotes income belongs to the lower 20% among the entire Korean population and supported by the Medical Aid program.
