# Supplementary material for: Association between exercise habits and stroke, heart failure, and mortality in Korean patients with incident atrial fibrillation: A nationwide population-based cohort study
Source: PLoS Med. 2021 Jun 8;18(6):e1003659. doi: 10.1371/journal.pmed.1003659 (PMC8219164; doi:10.1371/journal.pmed.1003659)
Supplement: S3 Table — ASD, absolute standardized difference; BMI, body mass index; BP, blood pressure; CKD, chronic kidney disease; COPD, chronic obstructive pulmonary disease; eGFR, estimated glomerular filtration rate; HDL, high-density lipoprotein; LDL, low-density lipoprotein; MI, myocardial infarction; OAC, oral anticoagulant; PAD, peripheral artery disease. *Group A denotes persistent non-exercisers, group B denotes new exercisers, group C denotes exercise dropouts, and group D denotes exercise maintainers. #Low income denotes income in the lowest 20% among the entire Korean population; individuals with low income are supported by the medical aid program. (DOCX) [file pmed.1003659.s005.docx]

**S3 Table**. The multiple comparisons of the baseline characteristics between the study groups presented as absolute standardized differences.

|  | ASD | | | | | | | | | | | | | |
| --- | --- | --- | --- | --- | --- | --- | --- | --- | --- | --- | --- | --- | --- | --- |
|  | Pre IPTW | | | | | | | Post IPTW | | | | | | |
|  | A* vs B* | A vs C* | A vs D* | B vs C | B vs D | C vs D | Maximum | A vs B | A vs C | A vs D | B vs C | B vs D | C vs D | Maximum |
| **Age** | 0.216 | 0.126 | 0.412 | 0.089 | 0.192 | 0.283 | 0.412 | 0.012 | 0.020 | 0.000 | 0.009 | 0.012 | 0.021 | 0.021 |
| **Sex** | 0.183 | 0.186 | 0.498 | 0.003 | 0.308 | 0.305 | 0.498 | 0.004 | 0.005 | 0.002 | 0.001 | 0.002 | 0.003 | 0.005 |
| Hypertension | 0.049 | 0.026 | 0.066 | 0.023 | 0.017 | 0.040 | 0.066 | 0.010 | 0.010 | 0.000 | 0.000 | 0.010 | 0.010 | 0.010 |
| Diabetes mellitus | 0.046 | 0.017 | 0.083 | 0.028 | 0.038 | 0.066 | 0.083 | 0.002 | 0.000 | 0.002 | 0.002 | 0.004 | 0.002 | 0.004 |
| Dyslipidemia | 0.014 | 0.001 | 0.021 | 0.014 | 0.007 | 0.022 | 0.022 | 0.002 | 0.001 | 0.002 | 0.001 | 0.000 | 0.001 | 0.002 |
| Previous MI | 0.027 | 0.018 | 0.030 | 0.009 | 0.003 | 0.012 | 0.030 | 0.002 | 0.001 | 0.001 | 0.001 | 0.003 | 0.002 | 0.003 |
| PAD | 0.055 | 0.019 | 0.143 | 0.036 | 0.088 | 0.123 | 0.143 | 0.005 | 0.003 | 0.001 | 0.002 | 0.006 | 0.005 | 0.006 |
| COPD | 0.065 | 0.062 | 0.164 | 0.004 | 0.098 | 0.102 | 0.164 | 0.001 | 0.002 | 0.001 | 0.001 | 0.000 | 0.002 | 0.002 |
| Cancer | 0.034 | 0.011 | 0.012 | 0.023 | 0.046 | 0.023 | 0.046 | 0.002 | 0.004 | 0.000 | 0.002 | 0.002 | 0.004 | 0.004 |
| CKD (GFR ≤ 60) | 0.099 | 0.065 | 0.137 | 0.034 | 0.038 | 0.072 | 0.137 | 0.006 | 0.005 | 0.001 | 0.001 | 0.007 | 0.006 | 0.007 |
| **CHA_2_DS_2_-VASc score** | 0.251 | 0.178 | 0.531 | 0.072 | 0.281 | 0.352 | 0.531 | 0.015 | 0.012 | 0.001 | 0.002 | 0.016 | 0.014 | 0.016 |
| **OAC** | 0.005 | 0.020 | 0.005 | 0.015 | 0.000 | 0.015 | 0.020 | 0.006 | 0.003 | 0.000 | 0.003 | 0.007 | 0.003 | 0.007 |
| Warfarin | 0.028 | 0.026 | 0.034 | 0.002 | 0.007 | 0.009 | 0.034 | 0.000 | 0.001 | 0.002 | 0.003 | 0.006 | 0.003 | 0.006 |
| NOAC | 0.039 | 0.004 | 0.055 | 0.035 | 0.016 | 0.051 | 0.055 | 0.003 | 0.003 | 0.003 | 0.000 | 0.006 | 0.006 | 0.006 |
| Aspirin | 0.044 | 0.014 | 0.029 | 0.030 | 0.016 | 0.014 | 0.044 | 0.007 | 0.005 | 0.000 | 0.002 | 0.008 | 0.005 | 0.008 |
| P2Y_12_ inhibitor | 0.028 | 0.020 | 0.039 | 0.008 | 0.011 | 0.019 | 0.039 | 0.000 | 0.001 | 0.004 | 0.001 | 0.005 | 0.004 | 0.005 |
| Statin | 0.038 | 0.033 | 0.056 | 0.004 | 0.018 | 0.022 | 0.056 | 0.002 | 0.001 | 0.001 | 0.001 | 0.001 | 0.000 | 0.002 |
| BMI (kg/m^2^) | 0.041 | 0.039 | 0.076 | 0.002 | 0.036 | 0.037 | 0.076 | 0.005 | 0.004 | 0.005 | 0.001 | 0.010 | 0.010 | 0.010 |
| Waist circumference (cm) | 0.013 | 0.046 | 0.063 | 0.033 | 0.051 | 0.017 | 0.063 | 0.030 | 0.010 | 0.076 | 0.020 | 0.047 | 0.067 | 0.076 |
| Systolic BP (mmHg) | 0.051 | 0.042 | 0.086 | 0.009 | 0.034 | 0.043 | 0.086 | 0.017 | 0.000 | 0.006 | 0.017 | 0.011 | 0.006 | 0.017 |
| Diastolic BP (mmHg) | 0.009 | 0.002 | 0.020 | 0.007 | 0.029 | 0.022 | 0.029 | 0.005 | 0.002 | 0.007 | 0.002 | 0.003 | 0.005 | 0.007 |
| Fasting glucose (mg/dL) | 0.026 | 0.001 | 0.029 | 0.026 | 0.002 | 0.029 | 0.029 | 0.003 | 0.003 | 0.003 | 0.006 | 0.000 | 0.006 | 0.006 |
| Total cholesterol (mg/dL) | 0.005 | 0.001 | 0.000 | 0.006 | 0.005 | 0.001 | 0.006 | 0.001 | 0.002 | 0.002 | 0.003 | 0.001 | 0.004 | 0.004 |
| eGFR (ml/min/1.73m^2^) | 0.050 | 0.022 | 0.034 | 0.027 | 0.018 | 0.011 | 0.050 | 0.027 | 0.024 | 0.068 | 0.002 | 0.038 | 0.041 | 0.068 |
| **Alcohol consumption  (Heavy Drinker)** | 0.005 | 0.015 | 0.063 | 0.009 | 0.058 | 0.048 | 0.063 | 0.004 | 0.003 | 0.001 | 0.001 | 0.003 | 0.002 | 0.004 |
| **Smoking** | 0.162 | 0.103 | 0.380 | 0.059 | 0.214 | 0.274 | 0.380 | 0.002 | 0.002 | 0.001 | 0.000 | 0.001 | 0.001 | 0.002 |
| **Low income ^#^** | 0.053 | 0.042 | 0.120 | 0.011 | 0.067 | 0.078 | 0.120 | 0.001 | 0.000 | 0.003 | 0.001 | 0.004 | 0.002 | 0.004 |

Abbreviation: ASD, absolute standardized difference; MI, myocardial infarction; PAD, peripheral artery disease; COPD, chronic obstructive pulmonary disease; CKD, chronic kidney disease; OAC, oral anticoagulant; BMI, body mass index; BP, blood pressure; LDL, low-density lipoprotein; HDL, high-density lipoprotein; eGFR, estimated glomerular filtration rate.

* Group A denotes persistent non-exerciser, B for new exerciser, C for exercise drop-outs, and D for exercise maintainer.

# Low income denotes income belongs to lower 20% among the entire Korean population and supported by the Medical Aid program.
