# Supplementary material for: Association between exercise habits and stroke, heart failure, and mortality in Korean patients with incident atrial fibrillation: A nationwide population-based cohort study
Source: PLoS Med. 2021 Jun 8;18(6):e1003659. doi: 10.1371/journal.pmed.1003659 (PMC8219164; doi:10.1371/journal.pmed.1003659)
Supplement: S4 Table — CI, confidence interval; HR, hazard ratio; IR, incidence rate; PY, person-years. *Major cardiovascular deaths are defined as deaths due to cerebral infarction (I63), acute myocardial infarction (I21), sequelae of cerebrovascular disease (I69), and heart failure (I50). Weighted event numbers and weighted IRs were computed after inverse probability of treatment weighting. The HRs were computed by weighted Cox proportional hazards models with inverse probability of treatment weighting. p-Values were evaluated by the likelihood ratio test. (DOCX) [file pmed.1003659.s006.docx]

**S4 Table.** Hazard ratios with 95% confidence intervals for major cardiovascular death according to the change of exercise status.

| **Major cardiovascular death *** | Number | Events | IR | HR |
| --- | --- | --- | --- | --- |
|  |  |  | (1000PY) | (95% CI) |
| Persistent non-exerciser | 20354 | 98.04 | 1.39 | 1 (Ref.) |
| New exerciser | 11874 | 41.90 | 1.02 | 0.74 (0.52-1.06) |
| Exercise drop-outs | 11630 | 39.67 | 0.98 | 0.71 (0.49-1.02) |
| Exercise maintainer | 22834 | 70.67 | 0.91 | 0.66 (0.49-0.90) |
|  |  |  |  | p = 0.040 |

Abbreviation: IR, incidence rate; PY, person-years; HR, hazard ratio; CI, confidence interval.

* Major cardiovascular deaths are defined as deaths due to cerebral infarction (I63), acute myocardial infarction (I21), sequelae of cerebrovascular disease (I69), and heart failure (I50).

Weighted event numbers and weighted IRs were computed after IPTW. The HRs were computed by weighted Cox proportional hazards models with IPTW.

*P* values were evaluated by the likelihood ratio test.
