# Supplementary material for: Association between exercise habits and stroke, heart failure, and mortality in Korean patients with incident atrial fibrillation: A nationwide population-based cohort study
Source: PLoS Med. 2021 Jun 8;18(6):e1003659. doi: 10.1371/journal.pmed.1003659 (PMC8219164; doi:10.1371/journal.pmed.1003659)
Supplement: S5 Table — Exercise dose presented as mean ± SD. CI, confidence interval; HR, hazard ratio; IR, incidence rate; MET, metabolic equivalent of task; PY, person-years. Model 1 adjusted for age and sex. Model 2 adjusted for age, sex, body mass index (BMI), hypertension, diabetes mellitus, dyslipidemia, previous myocardial infarction (MI), peripheral artery disease (PAD), chronic obstructive pulmonary disease (COPD), cancer, chronic kidney disease (CKD), CHA2DS2-VASc score, use of oral anticoagulation (OAC), use of antiplatelet agent, use of statin, smoking, heavy drinking, and low income. p-Values were evaluated by the likelihood ratio test. (DOCX) [file pmed.1003659.s007.docx]

**S5 Table.** Hazard ratios with 95% confidence intervals for ischemic stroke, heart failure, and all-cause death of new exerciser group (*No* to *Yes*) according to the energy expenditure (MET-min/wk).

|  | Exercise dose | Number | Events | IR (1000PY) | Unadjusted HR  (95% CI) | Model 1 HR  (95% CI) | Model 2 HR  (95% CI) |
| --- | --- | --- | --- | --- | --- | --- | --- |
| **Ischemic stroke** |  |  |  |  |  |  |  |
| Persistent non-exerciser | 187.4 ± 229.1 | 20354 | 707 | 10.36 | 1 (Ref.) | 1 (Ref.) | 1 (Ref.) |
| < 500 MET-min/wk | 332.1 ± 96.9 | 3207 | 93 | 8.37 | 0.81 (0.65-1.01) | 1.03 (0.83-1.28) | 1.04 (0.84-1.29) |
| 500-999 MET-min/wk | 727 ± 136.1 | 4585 | 118 | 7.66 | 0.74 (0.61-0.90) | 0.86 (0.71-1.05) | 0.88 (0.72-1.07) |
| 1000-1499 MET-min/wk | 1208.1 ± 159.7 | 2641 | 64 | 7.18 | 0.69 (0.54-0.90) | 0.76 (0.59-0.99) | 0.77 (0.60-0.99) |
| ≥ 1500 MET-min/wk | 1999.6 ± 325.3 | 1441 | 50 | 10.23 | 0.99 (0.74-1.32) | 0.96 (0.72-1.28) | 0.96 (0.72-1.28) |
|  |  |  |  |  | p = 0.002 | p = 0.180 | p = 0.229 |
| **Heart failure** |  |  |  |  |  |  |  |
| Persistent non-exerciser | 187.4 ± 229.1 | 20354 | 4365 | 71.86 | 1 (Ref.) | 1 (Ref.) | 1 (Ref.) |
| < 500 MET-min/wk | 332.1 ± 96.9 | 3207 | 601 | 59.86 | 0.84 (0.77-0.91) | 0.98 (0.90-1.07) | 0.98 (0.90-1.07) |
| 500-999 MET-min/wk | 727 ± 136.1 | 4585 | 858 | 61.64 | 0.86 (0.80-0.92) | 0.94 (0.88-1.02) | 0.95 (0.88-1.02) |
| 1000-1499 MET-min/wk | 1208.1 ± 159.7 | 2641 | 493 | 60.76 | 0.85 (0.77-0.93) | 0.89 (0.81-0.98) | 0.90 (0.82-0.99) |
| ≥ 1500 MET-min/wk | 1999.6 ± 325.3 | 1441 | 294 | 67.96 | 0.95 (0.84-1.06) | 0.91 (0.81-1.03) | 0.94 (0.83-1.06) |
|  |  |  |  |  | p < 0.001 | p = 0.069 | p = 0.166 |
| **All-cause death** |  |  |  |  |  |  |  |
| Persistent non-exerciser | 187.4 ± 229.1 | 20354 | 1119 | 16.04 | 1 (Ref.) | 1 (Ref.) | 1 (Ref.) |
| < 500 MET-min/wk | 332.1 ± 96.9 | 3207 | 130 | 11.48 | 0.71 (0.60-0.86) | 0.99 (0.83-1.19) | 1.02 (0.85-1.22) |
| 500-999 MET-min/wk | 727 ± 136.1 | 4585 | 175 | 11.17 | 0.70 (0.59-0.82) | 0.86 (0.73-1.01) | 0.90 (0.77-1.06) |
| 1000-1499 MET-min/wk | 1208.1 ± 159.7 | 2641 | 86 | 9.49 | 0.59 (0.48-0.74) | 0.67 (0.54-0.83) | 0.68 (0.54-0.84) |
| ≥ 1500 MET-min/wk | 1999.6 ± 325.3 | 1441 | 65 | 13.06 | 0.81 (0.63-1.04) | 0.76 (0.59-0.97) | 0.75 (0.58-0.96) |
|  |  |  |  |  | p < 0.001 | p < 0.001 | p = 0.002 |

Exercise dose presented as means ± SD.

Abbreviation: IR, incidence rate; PY, person-years; HR, hazard ratio; CI, confidence interval; MET, metabolic equivalent of task.

Model 1 adjusted for age and sex.

Model 2 adjusted for age, sex, body mass index (BMI), hypertension, diabetes mellitus, dyslipidemia, previous myocardial infarction (MI), peripheral artery disease (PAD), chronic obstructive pulmonary disease (COPD), cancer, chronic kidney disease (CKD), CHA_2_DS_2_-VASc score, use of oral anticoagulation (OAC), use of antiplatelet agents, use of statin, smoking, heavy drinking, and low income.

*P* values were evaluated by the likelihood ratio test.
