# Supplementary material for: Association between exercise habits and stroke, heart failure, and mortality in Korean patients with incident atrial fibrillation: A nationwide population-based cohort study
Source: PLoS Med. 2021 Jun 8;18(6):e1003659. doi: 10.1371/journal.pmed.1003659 (PMC8219164; doi:10.1371/journal.pmed.1003659)
Supplement: S6 Table — CI, confidence interval; HR, hazard ratio; IR, incidence rate; PY, person-years. Weighted event numbers and weighted IRs were computed after inverse probability of treatment weighting. The HRs were computed by weighted Cox proportional hazards models with inverse probability of treatment weighting. p-Values were evaluated by the likelihood ratio test. (DOCX) [file pmed.1003659.s008.docx]

**S6 Table.** Hazard ratios with 95% confidence intervals for ischemic stroke, heart failure, and all-cause death according to the change of exercise status and sex.

|  |  | Number | Events | IR (1000PY) | HR (95% CI) |
| --- | --- | --- | --- | --- | --- |
|  |  |  |  |  |  |
| **Ischemic stroke** | |  |  |  | *p-*for-interaction = 0.595 |
| **Male** | Persistent non -exerciser | 10676 | 433.54 | 9.82 | 1 (Ref.) |
|  | New exerciser | 7299 | 220.71 | 8.70 | 0.89 (0.75-1.04) |
|  | Exercise drop-outs | 7167 | 206.73 | 8.20 | 0.84 (0.71-0.99) |
|  | Exercise maintainer | 17268 | 403.59 | 8.30 | 0.84 (0.74-0.97) |
| **Female** | Persistent non-exerciser | 9678 | 199.68 | 7.92 | 1 (Ref.) |
|  | New exerciser | 4575 | 110.64 | 7.48 | 0.94 (0.75-1.19) |
|  | Exercise drop-outs | 4463 | 117.00 | 8.05 | 1.02 (0.81-1.28) |
|  | Exercise maintainer | 5566 | 193.63 | 7.09 | 0.89 (0.73-1.09) |
| **Heart failure** | |  |  |  | *p*-for-interaction = 0.371 |
| **Male** | Persistent non-exerciser | 10676 | 2743.67 | 69.59 | 1 (Ref.) |
|  | New exerciser | 7299 | 1500.31 | 65.86 | 0.95 (0.89-1.01) |
|  | Exercise drop-outs | 7167 | 1556.30 | 69.19 | 0.99 (0.93-1.06) |
|  | Exercise maintainer | 17268 | 2858.50 | 65.44 | 0.94 (0.89-0.99) |
| **Female** | Persistent non-exerciser | 9678 | 1388.64 | 61.18 | 1 (Ref.) |
|  | New exerciser | 4575 | 779.30 | 58.02 | 0.95 (0.87-1.04) |
|  | Exercise drop-outs | 4463 | 812.08 | 61.98 | 1.01 (0.93-1.11) |
|  | Exercise maintainer | 5566 | 1342.82 | 54.07 | 0.88 (0.82-0.95) |
| **All-cause death** | |  |  |  | *p*- for- interaction = 0.048 |
| **Male** | Persistent non-exerciser | 10676 | 761.05 | 16.87 | 1 (Ref.) |
|  | New exerciser | 7299 | 350.58 | 13.57 | 0.81 (0.71-0.91) |
|  | Exercise drop-outs | 7167 | 379.88 | 14.81 | 0.88 (0.78-0.99) |
|  | Exercise maintainer | 17268 | 490.78 | 9.90 | 0.59 (0.53-0.66) |
| **Female** | Persistent non-exerciser | 9678 | 220.14 | 8.59 | 1 (Ref.) |
|  | New exerciser | 4575 | 112.38 | 7.46 | 0.87 (0.69-1.09) |
|  | Exercise drop-outs | 4463 | 85.16 | 5.75 | 0.67 (0.52-0.86) |
|  | Exercise maintainer | 5566 | 160.29 | 5.77 | 0.68 (0.55-0.83) |

Abbreviation: IR, incidence rate; PY, person-years; HR, hazard ratio; CI, confidence interval.

Weighted event numbers and weighted IRs were computed after IPTW. The HRs were computed by weighted Cox proportional hazards models with IPTW.

*P* values were evaluated by the likelihood ratio test.
