# Supplementary material for: Association between exercise habits and stroke, heart failure, and mortality in Korean patients with incident atrial fibrillation: A nationwide population-based cohort study
Source: PLoS Med. 2021 Jun 8;18(6):e1003659. doi: 10.1371/journal.pmed.1003659 (PMC8219164; doi:10.1371/journal.pmed.1003659)
Supplement: S7 Table — CI, confidence interval; HR, hazard ratio; IR, incidence rate; PY, person-years. Weighted event numbers and weighted IRs were computed after inverse probability of treatment weighting. The HRs were computed by weighted Cox proportional hazards models with inverse probability of treatment weighting. p-Values were evaluated by the likelihood ratio test. (DOCX) [file pmed.1003659.s009.docx]

**S7 Table.** Hazard ratios with 95% confidence intervals for ischemic stroke, heart failure, and all-cause death according to the change of exercise status and age.

|  |  | Number | Events | IR (1000PY) | HR (95% CI) |
| --- | --- | --- | --- | --- | --- |
|  |  |  |  |  |  |
| **Ischemic stroke** | |  |  |  | *p*-for-interaction = 0.382 |
| **< 65 years** | Persistent non-exerciser | 10605 | 2076.01 | 5.32 | 1 (Ref.) |
|  | New exerciser | 7448 | 1148.75 | 4.86 | 0.91 (0.74-1.14) |
|  | Exercise drop-outs | 6787 | 1176.41 | 4.16 | 0.78 (0.62-0.98) |
|  | Exercise maintainer | 16376 | 2240.40 | 4.69 | 0.88 (0.74-1.06) |
| **65-74 years** | Persistent non-exerciser | 6508 | 1402.82 | 14.68 | 1 (Ref.) |
|  | New exerciser | 3270 | 789.09 | 11.89 | 0.81 (0.66-0.99) |
|  | Exercise drop-outs | 3539 | 833.11 | 13.96 | 0.95 (0.78-1.16) |
|  | Exercise maintainer | 5248 | 1472.91 | 11.92 | 0.81 (0.68-0.96) |
| **≥ 75 years** | Persistent non-exerciser | 3241 | 653.49 | 20.00 | 1 (Ref.) |
|  | New exerciser | 1156 | 341.77 | 21.76 | 1.09 (0.82-1.44) |
|  | Exercise drop-outs | 1304 | 358.86 | 18.83 | 0.94 (0.70-1.27) |
|  | Exercise maintainer | 1210 | 488.02 | 19.49 | 0.97 (0.76-1.26) |
| **Heart failure** | |  |  |  | *p*-for-interaction = 0.019 |
| **< 65 years** | Persistent non-exerciser | 10605 | 238.21 | 50.88 | 1 (Ref.) |
|  | New exerciser | 7448 | 125.14 | 48.47 | 0.95 (0.89-1.03) |
|  | Exercise drop-outs | 6787 | 105.13 | 51.03 | 1.00 (0.93-1.08) |
|  | Exercise maintainer | 16376 | 228.15 | 50.46 | 0.99 (0.93-1.05) |
| **65-74 years** | Persistent non-exerciser | 6508 | 264.74 | 88.85 | 1 (Ref.) |
|  | New exerciser | 3270 | 129.93 | 82.78 | 0.93 (0.85-1.02) |
|  | Exercise drop-outs | 3539 | 152.43 | 87.25 | 0.98 (0.90-1.07) |
|  | Exercise maintainer | 5248 | 257.68 | 77.00 | 0.87 (0.81-0.93) |
| **≥ 75 years** | Persistent non-exerciser | 3241 | 130.27 | 118.07 | 1 (Ref.) |
|  | New exerciser | 1156 | 76.27 | 114.74 | 0.97 (0.85-1.11) |
|  | Exercise drop-outs | 1304 | 66.16 | 119.77 | 1.01 (0.89-1.15) |
|  | Exercise maintainer | 1210 | 111.39 | 97.83 | 0.83 (0.74-0.93) |
| **All-cause death** | |  |  |  | *p*-for-interaction = 0.943 |
| **< 65 years** | Persistent non-exerciser | 10605 | 278.01 | 6.13 | 1 (Ref.) |
|  | New exerciser | 7448 | 127.45 | 4.89 | 0.80 (0.65-0.98) |
|  | Exercise drop-outs | 6787 | 135.19 | 5.29 | 0.86 (0.70-1.06) |
|  | Exercise maintainer | 16376 | 180.48 | 3.67 | 0.60 (0.50-0.72) |
| **65-74 years** | Persistent non-exerciser | 6508 | 372.47 | 19.98 | 1 (Ref.) |
|  | New exerciser | 3270 | 193.23 | 17.24 | 0.86 (0.73-1.03) |
|  | Exercise drop-outs | 3539 | 191.49 | 16.99 | 0.85 (0.71-1.01) |
|  | Exercise maintainer | 5248 | 285.04 | 12.80 | 0.64 (0.55-0.75) |
| **≥ 75 years** | Persistent non-exerciser | 3241 | 330.73 | 48.83 | 1 (Ref.) |
|  | New exerciser | 1156 | 142.27 | 38.97 | 0.81 (0.66-0.98) |
|  | Exercise drop-outs | 1304 | 138.37 | 37.77 | 0.77 (0.63-0.94) |
|  | Exercise maintainer | 1210 | 185.55 | 31.31 | 0.65 (0.54-0.77) |

Abbreviation: IR, incidence rate; PY, person-years; HR, hazard ratio; CI, confidence interval.

Weighted event numbers and weighted IRs were computed after IPTW. The HRs were computed by weighted Cox proportional hazards models with IPTW.

*P* values were evaluated by the likelihood ratio test.
