# Supplementary material for: Association between exercise habits and stroke, heart failure, and mortality in Korean patients with incident atrial fibrillation: A nationwide population-based cohort study
Source: PLoS Med. 2021 Jun 8;18(6):e1003659. doi: 10.1371/journal.pmed.1003659 (PMC8219164; doi:10.1371/journal.pmed.1003659)
Supplement: S8 Table — CI, confidence interval; HR, hazard ratio; IR, incidence rate; PY, person-years. Weighted event numbers and weighted IRs were computed after inverse probability of treatment weighting. The HRs were computed by weighted Cox proportional hazards models with inverse probability of treatment weighting. p-Values were evaluated by the likelihood ratio test. (DOCX) [file pmed.1003659.s010.docx]

**S8 Table.** Hazard ratios with 95% confidence intervals for ischemic stroke, heart failure, and all-cause death according to the change of exercise status and CHA_2_DS_2_-VASc score.

|  |  | Number | Events | IR (1000PY) | HR (95% CI) |
| --- | --- | --- | --- | --- | --- |
|  |  |  |  |  |  |
| **Ischemic stroke** |  |  |  |  | *p*-for-interaction = 0.699 |
| **CHA_2_DS_2_-VASc < 3** | Persistent non-exerciser | 12289 | 342.15 | 6.83 | 1 (Ref.) |
|  | New exerciser | 8431 | 171.48 | 5.91 | 0.87 (0.72-1.04) |
|  | Exercise drop-outs | 7903 | 161.89 | 5.65 | 0.83 (0.69-0.99) |
|  | Exercise maintainer | 18404 | 317.87 | 5.78 | 0.85 (0.73-0.98) |
| **CHA_2_DS_2_-VASc ≥ 3** | Persistent non-exerciser | 8065 | 291.08 | 15.11 | 1 (Ref.) |
|  | New exerciser | 3443 | 159.87 | 14.33 | 0.95 (0.78-1.15) |
|  | Exercise drop-outs | 3727 | 161.84 | 14.60 | 0.97 (0.80-1.17) |
|  | Exercise maintainer | 4430 | 279.35 | 13.35 | 0.88 (0.75-1.04) |
| **Heart failure** |  |  |  |  | *p*-for-interaction = 0.108 |
| **CHA_2_DS_2_-VASc < 3** | Persistent non-exerciser | 12289 | 2578.78 | 56.75 | 1 (Ref.) |
|  | New exerciser | 8431 | 1376.39 | 51.73 | 0.91 (0.85-0.97) |
|  | Exercise drop-outs | 7903 | 1469.98 | 56.64 | 0.99 (0.94-1.06) |
|  | Exercise maintainer | 18404 | 2657.33 | 52.91 | 0.93 (0.88-0.98) |
| **CHA_2_DS_2_-VASc ≥ 3** | Persistent non-exerciser | 8065 | 1553.54 | 93.09 | 1 (Ref.) |
|  | New exerciser | 3443 | 903.22 | 94.03 | 1.01 (0.93-1.10) |
|  | Exercise drop-outs | 3727 | 898.39 | 93.16 | 1.00 (0.92-1.09) |
|  | Exercise maintainer | 4430 | 1544.00 | 84.41 | 0.91 (0.85-0.97) |
| **All-cause death** |  |  |  |  | *p*-for-interaction = 0.329 |
| **CHA_2_DS_2_-VASc < 3** | Persistent non-exerciser | 12289 | 495.53 | 9.75 | 1 (Ref.) |
|  | New exerciser | 8431 | 231.08 | 7.86 | 0.81 (0.69-0.94) |
|  | Exercise drop-outs | 7903 | 238.95 | 8.24 | 0.85 (0.73-0.99) |
|  | Exercise maintainer | 18404 | 305.18 | 5.47 | 0.56 (0.49-0.65) |
| **CHA_2_DS_2_-VASc ≥ 3** | Persistent non-exerciser | 8065 | 485.67 | 24.40 | 1(Ref.) |
|  | New exerciser | 3443 | 231.89 | 20.17 | 0.83 (0.71-0.97) |
|  | Exercise drop-outs | 3727 | 226.09 | 19.71 | 0.81 (0.69-0.94) |
|  | Exercise maintainer | 4430 | 345.89 | 16.05 | 0.66 (0.57-0.76) |

Abbreviation: IR, incidence rate; PY, person-years; HR, hazard ratio; CI, confidence interval.

Weighted event numbers and weighted IRs were computed after IPTW. The HRs were computed by weighted Cox proportional hazards models with IPTW.

*P* values were evaluated by the likelihood ratio test.
