# Supplementary material for: Association between exercise habits and stroke, heart failure, and mortality in Korean patients with incident atrial fibrillation: A nationwide population-based cohort study
Source: PLoS Med. 2021 Jun 8;18(6):e1003659. doi: 10.1371/journal.pmed.1003659 (PMC8219164; doi:10.1371/journal.pmed.1003659)
Supplement: S9 Table — CI, confidence interval; HR, hazard ratio; IR, incidence rate; PY, person-years. Model 1 adjusted for age and sex. Model 2 adjusted for age, sex, body mass index (BMI), hypertension, diabetes mellitus, dyslipidemia, previous myocardial infarction (MI), peripheral artery disease (PAD), chronic obstructive pulmonary disease (COPD), cancer, chronic kidney disease (CKD), CHA2DS2-VASc score, use of oral anticoagulation (OAC), use of antiplatelet agent, use of statin, smoking, heavy drinking, and low income. Model 3 adjusted for age, sex, BMI, hypertension, diabetes mellitus, dyslipidemia, previous MI, PAD, COPD, cancer, CKD, CHA2DS2-VASc score, use of OAC, use of antiplatelet agent, use of statin, smoking, heavy drinking, low income, and Charlson Comorbidity Index (CCI). p-Values were evaluated by the likelihood ratio test. (DOCX) [file pmed.1003659.s011.docx]

**S9 Table.** Hazard ratios with 95% confidence intervals for ischemic stroke, heart failure, and all-cause death according to the change of exercise status calculated from the multivariable-adjusted Cox proportional hazard model.

|  | Number | Events | IR  (1000PY) | Unadjusted HR  (95% CI) | Model 1 HR  (95% CI) | Model 2 HR  (95% CI) | Model 3 HR  (95% CI) |
| --- | --- | --- | --- | --- | --- | --- | --- |
| **Ischemic stroke** |  |  |  |  |  |  |  |
| Persistent non-exerciser | 20354 | 707 | 10.36 | 1 (Ref.) | 1 (Ref.) | 1 (Ref.) | 1 (Ref.) |
| New exerciser | 11874 | 325 | 8.06 | 0.78 (0.68-0.89) | 0.90 (0.79-1.03) | 0.92 (0.80-1.05) | 0.92 (0.80-1.05) |
| Exercise drop-outs | 11630 | 339 | 8.58 | 0.83 (0.73-0.94) | 0.90 (0.79-1.02) | 0.91 (0.79-1.03) | 0.91 (0.80-1.03) |
| Exercise maintainer | 22834 | 534 | 6.94 | 0.67 (0.60-0.75) | 0.88 (0.78-0.99) | 0.89 (0.79-1.00) | 0.89 (0.79-1.00) |
|  |  |  |  | p < 0.001 | p = 0.127 | p = 0.203 | p = 0.216 |
| **Heart failure** |  |  |  |  |  |  |  |
| Persistent non-exerciser | 20354 | 4365 | 71.86 | 1 (Ref.) | 1 (Ref.) | 1 (Ref.) | 1 (Ref.) |
| New exerciser | 11874 | 2246 | 61.71 | 0.86 (0.82-0.91) | 0.93 (0.88-0.98) | 0.94 (0.89-0.99) | 0.94 (0.89-0.99) |
| Exercise drop-outs | 11630 | 2420 | 68.51 | 0.95 (0.91-1.00) | 0.99 (0.94-1.04) | 1.00 (0.95-1.05) | 1.00 (0.95-1.05) |
| Exercise maintainer | 22834 | 4081 | 58.53 | 0.82 (0.78-0.85) | 0.93 (0.89-0.97) | 0.93 (0.89-0.98) | 0.94 (0.90-0.98) |
|  |  |  |  | p < 0.001 | p = 0.002 | p = 0.017 | p = 0.007 |
| **All-cause death** |  |  |  |  |  |  |  |
| Persistent non-exerciser | 20354 | 1119 | 16.04 | 1 (Ref.) | 1 (Ref.) | 1 (Ref.) | 1 (Ref.) |
| New exerciser | 11874 | 456 | 11.11 | 0.69 (0.62-0.77) | 0.82 (0.74-0.92) | 0.84 (0.75-0.94) | 0.83 (0.75-0.93) |
| Exercise drop-outs | 11630 | 489 | 12.13 | 0.76 (0.68-0.84) | 0.81 (0.73-0.90) | 0.83 (0.75-0.93) | 0.84 (0.75-0.93) |
| Exercise maintainer | 22834 | 563 | 7.19 | 0.45 (0.41-0.50) | 0.60 (0.54-0.67) | 0.63 (0.57-0.70) | 0.65 (0.58-0.72) |
|  |  |  |  | p < 0.001 | p < 0.001 | p < 0.001 | p < 0.001 |

Abbreviation: IR, incidence rate; PY, person-years; HR, hazard ratio; CI, confidence interval.

Model 1 adjusted for age and sex.

Model 2 adjusted for age, sex, body mass index (BMI), hypertension, diabetes mellitus, dyslipidemia, previous myocardial infarction (MI), peripheral artery disease (PAD), chronic obstructive pulmonary disease (COPD), cancer, chronic kidney disease (CKD), CHA_2_DS_2_-VASc score, use of oral anticoagulation (OAC), use of antiplatelet agents, use of statin, smoking, heavy drinking, and low income.

Model 3 adjusted for age, sex, body mass index (BMI), hypertension, diabetes mellitus, dyslipidemia, previous myocardial infarction (MI), peripheral artery disease (PAD), chronic obstructive pulmonary disease (COPD), cancer, chronic kidney disease (CKD), CHA_2_DS_2_-VASc score, use of oral anticoagulation (OAC), use of antiplatelet agents, use of statin, smoking, heavy drinking, low income, and Charlson Comorbidity Index (CCI).

*P* values were evaluated by the likelihood ratio test.
