# Supplementary material for: Association between exercise habits and stroke, heart failure, and mortality in Korean patients with incident atrial fibrillation: A nationwide population-based cohort study
Source: PLoS Med. 2021 Jun 8;18(6):e1003659. doi: 10.1371/journal.pmed.1003659 (PMC8219164; doi:10.1371/journal.pmed.1003659)
Supplement: Attachment — Submitted filename: [Exercise_CVD_AF] Response to review_final.docx [file pmed.1003659.s013.docx]

**Requests from the editors:**

***In the last sentence of the Abstract Methods and Findings section, please include a brief note about any key limitation(s) of the study's methodology.**

**Response**: Thank you for your comments. As requested, we have described the key limitations of the study’s methodology in the last part of the Abstract Methods and Findings section.

*Abstract (Page 2, Lines 46-49)*

Recall bias might be introduced due to the nature of the self-reported questionnaire and external generalizability is limited, particularly to western populations, since our study was conducted in an Asian population.

***At this stage, we ask that you include a short, non-technical Author Summary of your research to make findings accessible to a wide audience that includes both scientists and non-scientists. The Author Summary should immediately follow the Abstract in your revised manuscript. This text is subject to editorial change and should be distinct from the scientific abstract. Please see our author guidelines for more information: https://journals.plos.org/plosmedicine/s/revising-your-manuscript#loc-author-summary**

**Response**: Thank you for your comments. As requested, we have described the Author Summary of our research after the Abstract section.

*Author summary (Page 4-5, Lines 55-85)*

**Why Was This Study Done?**

Atrial fibrillation (AF) is associated with an increased risk of stroke, heart failure, myocardial infarction, dementia, and death. As AF and its related health care burden are expected to surge, an integrated approach to the management of AF is an essential part of care.

Exercise has been established to benefit on AF related outcomes, including symptoms, incidence, recurrence, burden, and quality of life. However, the specific influence of exercise on cardiovascular morbidities in patients with AF is unknown.

**What Did the Researchers Do and Find?**

This is a nationwide population-based cohort study using data from the Korea National Health Insurance Service. We performed a retrospective analysis of 66 692 patients with newly diagnosed AF who underwent two serial health examinations before and after their AF diagnosis from 2010 to 2016.

Exercise status was evaluated by a self-reported questionnaire in each health examination, and the incidences of ischemic stroke, heart failure, and all-cause death were compared across the groups categorized according to the change of exercise status.

Patients who initiated or continued regular exercise after a diagnosis of AF were associated with a 6%-10% lower risk of HF and 16%-37% lower risk of mortality compared to persistent non-exerciser. With respect to ischemic stroke, the estimates of hazard ratios were 8%-11% lower in patients with the exercise group, yet statistical significance was undetermined.

In addition, regular moderate exercise 170-240 min/week was associated with the lowest risk of cardiovascular outcomes in patients who initiate exercise after the diagnosis of AF.

**What Do These Findings Mean?**

AF and AF-related complications confer a major healthcare burden. Thus lifestyle modifications are advocated as part of holistic management in current AF management guidelines.

As a lifestyle intervention, our result supports evidence of exercise benefits in cardiovascular outcomes that are cost-effective and safe. Also, our findings suggest a specific volume of exercise (regular moderate exercise 170-240 min/week). Encouragement of exercise should be recommended in AF patients to lower the future risk of adverse cardiovascular outcomes.

***Please clarify if the analytical approach reported here corresponded to one laid out in a prospective protocol or analysis plan? Please state this (either way) early in the Methods section.
a) If a prospective analysis plan (from your funding proposal, IRB or other ethics committee submission, study protocol, or other planning document written before analyzing the data) was used in designing the study, please include the relevant prospectively written document with your revised manuscript as a Supporting Information file to be published alongside your study, and cite it in the Methods section. A legend for this file should be included at the end of your manuscript.
b) If no such document exists, please make sure that the Methods section transparently describes when analyses were planned, and when/why any data-driven changes to analyses took place.
c) In either case, changes in the analysis-- including those made in response to peer review comments-- should be identified as such in the Methods section of the paper, with rationale.**

**Response:** Thank you for your comments. As requested, we had described when data analyses were performed and additional analysis in response to peer review comments in the Method section.

*Method (Page 10, Lines 179-181)*

Data collection and statistical analysis were performed using SAS version 9.4 (SAS Institute, Cary, NC) from January 2020 to September 2020.

*Method (Page 10, Lines 185-191)*

In addition, we analyzed the causes of death to scrutinize the positive risk-benefit of all-cause death using the ICD-10 code claimed at death. The most frequent four major specific disease entities, which belong to diseases of the circulatory system (I-code in ICD-10 system) and explain the cause of death among Korean AF patients, were used to define major cardiovascular death: cerebral infarction (I63), acute myocardial infarction (I21), sequelae of cerebrovascular disease (I69), and heart failure (I50) [14]. Second, we further adjusted primary outcomes by comorbidities or frailty using the Charlson Comorbidity Index (CCI) along with Model 2.

***We'd suggest ensuring that the study is reported according to the STROBE guideline, and the completed STROBE checklist should be included as Supporting Information with the revised paper. Please add the following statement, or similar, to the Methods: "This study is reported as per the Strengthening the Reporting of Observational Studies in Epidemiology (STROBE) guideline (S1 Checklist)." The STROBE guideline can be found here: http://www.equator-network.org/reporting-guidelines/strobe/. When completing the checklist, please use section and paragraph numbers, rather than page numbers.**

**Response:** Thank you for your comments. We added the requested statement in the Methods

*Method (Page 7, Lines 116-118)*

This study is reported as per the Strengthening the Reporting of Observational Studies in Epidemiology (STROBE) guideline (S1 Checklist).

**Reviewer #1: The study by Ahn and coworkers provides "big data" from nationwide population based cohort study on the impact of exercise on clinical sequelae of newly diagnosed atrial fibrillation.**

**They retrieved data from more than 66'000 South Korean patients how had their first diagnosis of atrial fibrillation between two consecutive biennially health care examinations from the National Health Insurance data.**

**The categorized these patients according to their exercise behavior before and after the diagnosis into 4 groups. The primary endpoint was the incidence of stroke, heart failure and overall mortality. Furthermore, they strived to identify the optimal amount of exercise per week in order to decrease the clinical endpoints in patients who newly started to exercise.**

**The paper is very well written and the analysis is based on an impressive number of patients. The results are relevant since they underline the importance of initiating or maintaining regular exercise in order to decrease the incidence of heart failure and overall mortality. Furthermore, it provides some guidance as to the weekly duration of moderate exercise in order to derive the maximum benefit.**

**Response:** We greatly appreciate your valuable comment and concern.

**Specific comments:**

**It needs to be mentioned in the limitation section that these results apply to an Asian population which shows some physiological as well as cultural differences.**

**Response:** Thank you for your comments. We mentioned the limited external generalizability of our results, particularly to Western populations, in the Limitations section.

*Discussion (Page 18, Linse 353-355)*

Sixth, the study was conducted in an Asian population; thereby, physiological as well as cultural differences in exercise behavior and biological response to exercise might limit external generalizability, particularly to western populations.

**The amount of women who do not exercise at all appears to be much higher than in a Western European population.**

**Response**: Thank you. Previous studies from Western populations have reported that the prevalence of non-exercisers in females was 34% to 47.2% [28, 29]. The HUNT3 study, which was based on population data of Norway, reported 38.6% of inactive patients with AF were women [28]. Also, among female AF patients, 34.0% were categorized as inactive in exercise. The EORP-AF registry reported that 48.7% of the no physical activity group were women, and 47.2% of female AF patients were categorized into the no physical activity group [29].

In our study, 47.6% of persistent non-exercisers were women, and 39.9% of the total female AF patients were categorized as a persistent non-exerciser group. Hence, this proportion of inactive women was comparable with the values reported in the HUNT3 study and the EORP-AF registry; indeed, our study population does not represent a male-dominant AF patient population. However, there could exist cultural difference which contributes to various proportions of reported inactive female AF patients among the different ethnic groups.

*Added Reference*

28.	Garnvik LE, Malmo V, Janszky I, Ellekjaer H, Wisloff U, Loennechen JP, et al. Physical activity, cardiorespiratory fitness, and cardiovascular outcomes in individuals with atrial fibrillation: the HUNT study. Eur Heart J. 2020. Epub 2020/02/13. doi: 10.1093/eurheartj/ehaa032. PubMed PMID: 32047884.

29.	Proietti M, Boriani G, Laroche C, Diemberger I, Popescu MI, Rasmussen LH, et al. Self-reported physical activity and major adverse events in patients with atrial fibrillation: a report from the EURObservational Research Programme Pilot Survey on Atrial Fibrillation (EORP-AF) General Registry. Europace. 2017;19(4):535-43. Epub 2017/04/22. doi: 10.1093/europace/euw150. PubMed PMID: 28431068.

**One of the biggest drawbacks is certainly that we do not have any information on the type and burden of AF.**

**Response:** Thank you. Yes, this is one of the common limitations among research using data provided by national healthcare service and insurance. We have mentioned this point in the Discussion section.

*Discussion (Page 18, Lines 350-352)*

Fourth, the type and burden of AF were not available; thus, the impact of these factors on the outcomes could not be analyzed.

**Reviewer #2: This is a statistical review of manuscript PMEDICINE-D-20-03103_R1. The manuscript is well-written, well-structured and I must say that Figure 1 is really good because it is easy to understand and it captures the whole study design. I have one question for the authors, two suggestions and a few minor points.**

**Question:**

**1/ "Lower risks of ischemic stroke, HF, and all-cause death were correlated with energy expenditure increment, but with a trend towards increased risks at ≥1500 MET min/week group". In eTable2, I've noticed that the sample size for the group >= 1500 is the smallest by far. Is there any biological plausibility for the U-shape for the HR curve in Figure 3? The CIs overlap so much that I am not sure one can conclude and mention in the abstract that 1000-1499 is the "best" amount of exercise. What are your thoughts? I would suggest to perhaps tone down the conclusions with respect to this analysis. If anything, I see a downward trend.**

**Response:** We appreciate your valuable comments and concerns. We agree with your comment that the limited number of those with more than 1500 MET min/week might have influenced the statistical analyses. It is well-known that excessive exercise would increase the risk of AF, possibly due to autonomic activation, atrial dilation, and fibrosis [45]. Hence there is some biological plausibility, but given the small numbers, we have added text to the Discussion section regarding the results of those with more than 1500 MET min/week.

We found that those with 1000-1499 METs-min/week (regular moderate exercise 170-240 min/week) showed the lowest risk of stroke, heart failure, and death in the new-exercisers group. Although this value does not imply the “best” cut-off values of exercise amount to prevent adverse clinical outcomes, we mention that this range has the maximum benefit of exercise seen on stroke, heart failure, and death.

*Discussion (Page 16, Lines 315-318)*

However, considering the wide 95% CIs in patients with ≥1500 MET-min/week, the small patient numbers in this group might be underpowered, therefore further research would be required to delineate the optimal range of exercise levels that could derive the greatest benefit among cardiovascular outcomes in patients with AF.

*Added Reference*

45.	Morseth B, Lochen ML, Ariansen I, Myrstad M, Thelle DS. The ambiguity of physical activity, exercise and atrial fibrillation. Eur J Prev Cardiol. 2018;25(6):624-36. Epub 2018/02/08. doi: 10.1177/2047487318754930. PubMed PMID: 29411631.

**Suggestions:**

**1/ the HRs that you report in the abstract are adjusted HRs. I think that this fact could be mentioned, e.g adjusted hazard ratio (HR) 0.94**

**Response:** Thank you. We have revised the text following your comment.

**2/ we performed reexamination of outcomes within subgroups of sex, age, and CHA2DS2-VASc score. I think it would be helpful to provide more details on the model. Was the model the same as Model 2, with an interaction term between exercise category and sex, age or the score?**

**Response:** The model was the same as model 2, except for each interaction term. We revised the description in the Methods.

*Method (Page 10, Lines 183-184)*

We performed a subgroup analysis of primary outcomes according to sex, age, and CHA_2_DS_2_-VASc score using the aforementioned Model 2 with each interaction factor.

**Minor points: I've noticed two sentences where I think one word was missing (see [xxx])**

**1/ The amount of energy expenditure [that] maximizes cardiovascular benefits appears to be**

**2/ Since we evaluated de novo incidence of ischemic stroke and HF after AF diagnosis, patients [with] recorded prior history of ischemic stroke and HF before the index date (health examination date after AF diagnosis) were excluded**

**Response:** Thank you. We have revised the typos.

**Reviewer #3: The study by Ahn et al sought to determine the influence of exercise after atrial fibrillation (AF) diagnosis on risk of stroke, heart failure and all-cause death. The topic and aim are highly relevant given that specific exercise guidelines are lacking for AF patients and there is a paucity of data on cardiovascular outcomes after exercise in established AF. Particularly novel is the use of data on exercise habits before and after first AF diagnosis. The design and results strengthen the idea that exercise is causally related to outcomes, in a relatively short term, and therefore might have a place in secondary prevention and cardiac rehabilitation of these patients. Other strengths of the study are a large population sample, a high event rate and seemingly valid and relevant endpoints. The paper is also well-written and structured. I have only some minor comments that may improve the manuscript:**

*** Stroke risk is obviously a major issue in AF and exercise may play a role in risk reduction. Unfortunately, this study, similar to the cited study from the HUNT cohort, was not able to provide convincing evidence that exercise adoption or maintenance would greatly reduce the risk of stroke. Still I think the statement that `exercise was associated lower risk of HF, but not stroke` (abstract, conclusion etc.) is too strong and may even be misleading when read briefly and out of context (as many do). The main effect estimates (HR) for stroke in exercisers actually seem in line with, or even stronger, than those for HF although precision obviously is lower. Whether the lack of precision is due to power (events) or other confounders/effect modifiers could be discussed. Also, a certain amount of exercise in new exercisers were even "significant" (Fig 3/eTable 2). I would consider re-wording these conclusions in a neutral language, including line 219 ("impact..not as strong") given that this may be the take-home-message for many readers. Keeping the left x-axis in Fig. 2 consistent across outcomes would also help on correct interpretation.**

**Response:** We greatly appreciate your comments and concerns. We have revised the descriptions regarding the result of ischemic stroke and updated **Fig 2**.

*Abstract (Page 3, Lines 50-51)*

Patients who initiated or continued regular exercise after a diagnosis of AF were associated with a lower risk of HF and mortality.

*Result (Page 12, Lines 222-225)*

For ischemic stroke, participating in exercise was associated with 8-11% lower risk compared to the persistent non-exerciser group, but the precision in the estimates of adjusted HRs was statistically insignificant: adjusted HR (95% CI) 0.92 (0.80-1.05) for the new exerciser group, and 0.89 (0.79-1.00) for exercise maintainer group.

*Discussion (Page 15, Linse 293-294)*

On the other hand, we found that the association of exercise and the risk of stroke was statistically insignificant.

*Discussion (Page 16, Linse 301-303)*

Relatively low event rate of ischemic stroke compared to HF and all-cause death might attribute to the lack of statistical power; thus, further research needs to address the association between exercise and the risk of ischemic stroke.

*Conclusion (Page 18, Lines 368-369)*

In this nationwide population-based cohort study, patients who initiated or continued regular exercise after a diagnosis of AF were associated with lower risks of HF and mortality.

*** Similarly, at p. 9, line 176, it is stated that the highest exercise volumes had a "trend towards increased risks", although the main effect were even lower than the reference, even significantly for deaths. Although I understand the statement refer to the J-shape (or reverse J-shape?) and a comparison to the second highest category, I suggest change to "attenuated risk reduction" or similar.**

**Response:** We greatly appreciate your valuable comments. We have revised the description regarding the analysis of energy expenditure following your comments

*Result (Page 12, Lines 228-235)*

Exercising 1000-1499 MET-min/week (1208.1 ± 159.7 MET-min/week) was consistently associated with a lower risk of adverse cardiovascular outcomes (adjusted HR 0.77, 95% CI 0.60-0.99 for ischemic stroke and adjusted HR 0.90, 95% CI 0.82-0.99 for HF) and all-cause death (adjusted HR 0.68, 95% CI 0.54-0.84) compared to persistent non-exerciser. Lower risks of ischemic stroke, HF, and all-cause death were correlated with energy expenditure increment, but the associations became attenuated in the group with exercise amount above 1500 MET-min/week (**Fig 3**).

*Discussion (Page 16, Lines 307-314)*

In the present study, we found that AF patients exercising 1000-1499 MET-min/week were commonly associated with the lower event rates for stroke, HF, and mortality in the new exerciser group. Interestingly, the association appeared to be attenuated in the range of exercise ≥1500 MET-min/week rather than persistently showing a direct inverse correlation. The mechanisms of intensive exercise to increase the risk of AF are diverse, including changes in autonomic activation [46, 47], atrial dilatation [48, 49], fibrosis, and inflammation [50, 51]. Similar physiologic changes would explain the attenuated risk-benefit in AF patients with exercise ≥1500 MET-min/week across cardiovascular outcomes, particularly in all-cause death.

*** PA was reported ~1 year before and after AF diagnosis and that is a strength of the study, but might also be a limitation. I.e. a potential change in PA status from examination to end of follow up might bias the associations and is not mentioned in limitations. In line with that, I`m surprised about the number of "new exercisers", compared to drop-outs after being diagnosed with this exercise-limiting disease (by the way, I would change the word "drop-out" just for the reasons above, as well as "any lifetime experience" at line 188). Can you just comment? Are there any exercise recommendations/rehabilitation standards after AF diagnosis in Korea?**

**Response:** Thank you for the comments. We agree that a possible change in exercise status after the index date might affect the risk of AF. We now state the aspects in the Limitation section.

*Discussion (Page 18, Lines 352-353)*

Fifth, potential change in exercise status from the index date to the end of follow up might introduce bias in the associations

Since we compared exercise status before and after AF diagnosis, we chose the word “drop-out” to reflect the change from exercise “Yes” at the health examination before AF diagnosis to “No” at the exam after AF diagnosis. Considering the word length and convenience to refer to within the article, we would prefer to keep this term. We clarified the definition of each group in the Method section.

*Method (Page 8, Lines 140-144)*

By comparing categorization of exercise status at each health examination before and after AF diagnosis, the study population was divided into four groups: persistent non-exerciser (***No*** to ***No***), new exerciser (***No*** to ***Yes***), exercise drop-outs (***Yes*** to ***No***), and exercise maintainer (***Yes*** to ***Yes***). **Fig 1B** summarizes the overall configuration of our study and the selection of the population included in the analysis.

Moreover, we deleted the term “any lifetime experience” patients, which meant new exercisers, exercise drop-outs, and exercise maintainers together, to avoid confusion in the context of a possible change of exercise status after the index date. Instead, we stated the respective group or revised an expression in the sentences that mentioned “any lifetime experience.”

*Abstract (Page 2, Line 40-44)*

Also, patients performing exercise any time before or after AF diagnosis were associated with a lower risk of mortality compared to the persistent non-exerciser; adjusted HR (95% CI) was 0.84 (0.75-0.94) for new exerciser, 0.83 (0.75-0.93) for exercise drop-outs, and 0.63 (0.57-0.70) for exercise maintainer.

*Discussion (Page 14, Line 256-260)*

First, initiation and maintenance of exercise after AF diagnosis was associated with a significantly lower risk for HF and all-cause death. Interestingly, exercise drop-outs were associated with a lower risk of all-cause death, as well. Second, the estimates of HRs of ischemic stroke were lower in AF patients with exercise, though the association was statistically insignificant.

We have our own guideline about optimal management of patients with AF incorporating data of the Korean population (Korean Circ J. 2018;48(12):1033-80.). However, a specific recommendation about the intensity and duration of exercise was not provided as recently updated AF guideline [31]. Nevertheless, newly diagnosed AF patients might initiate exercise by virtue of physicians’ emphasis on lifestyle modifications, thus contribute to the considerable number of new exerciser. To the best of our knowledge, this is the first study that suggests the specific amount of exercise that could bring benefits in cardiovascular outcomes with statistical significance.

*Added Reference*

31.	Hindricks G, Potpara T, Dagres N, Arbelo E, Bax JJ, Blomstrom-Lundqvist C, et al. 2020 ESC Guidelines for the diagnosis and management of atrial fibrillation developed in collaboration with the European Association of Cardio-Thoracic Surgery (EACTS). Eur Heart J. 2020. Epub 2020/08/30. doi: 10.1093/eurheartj/ehaa612. PubMed PMID: 32860505.

*** The secondary analyses on energy expenditure is interesting and provides novel information for future guidelines. But why were only new exercisers analysed? Would there be differences in maintainers? I think "adopters" or "new exercisers" could be added to the sentence at lines 189-90 since this conclusion about volumes is only valid for previously inactive patients` exercise a year after diagnosis.**

**Response:** Thank you. We tried to analyze energy expenditure, especially in the new exerciser group, to estimate the influence of exercise per se (exercise introduction) by isolating other healthy confounders that may attribute to exercise maintenance. In the group with exercise maintainer, the most beneficial range of exercise volume was different among primary endpoints (**eTable9 in the Supplement**). In this analysis, the energy expenditure 1000-1499 MET-min/wk had the most beneficial range of exercise volume in the aspect of all-cause death. However, this range was different for the outcomes of ischemic stroke and heart failure. The energy expenditure 500-999 Met-min/wk was a range with the lowest risk of ischemic stroke, and exercising ≥1500 MET-min/wk was associated with the lowest risk of heart failure. We assumed that the effect of accumulated exercise would have an influence on the clinical outcomes with different impacts.

We provide evidence of a dose amount of exercise in those who started exercise after newly diagnosed with AF. We hope this evidence would be incorporated into future guidelines. We have revised the conclusion about energy volume by stating that 1000-1499 MET-min/wk was associated with the lowest risks of ischemic stroke, heart failure, and all-cause death *in the “new exerciser” group*.

*Abstract (Page 2, Lines 44-46)*

Energy expenditure 1000-1499 metabolic equivalents of task-min/week (regular moderate exercise 170-240 min/week) was associated with a lower risk of each outcome based on a subgroup analysis of the new exerciser group.

*Methods (Page 8, Lines 147-149)*

This analysis was performed in the new exerciser (***No*** to ***Yes***) group to measure the influence of exercise introduction by isolating other healthy confounders that might attribute to maintenance of exercise.

*Discussion (Page 14, Lines 260-263)*

Third, performing exercise 1000-1499 MET-min/week (moderate-intensity exercise 170-240 min/week or vigorous-intensity exercise 140-210 min/week) was associated with lower risks of ischemic stroke, HF, and all-cause death in the new exerciser group.

*Discussion (Page 16, Lines 307-309)*

In the present study, we found that AF patients exercising 1000-1499 MET-min/week were associated with the lower event rates for stroke, HF, and mortality in the new exerciser group

*Conclusion (Page 19, Lines 369-371)*

Regular moderate exercise 170-240 min/week was associated with a favorable risk-benefit across cardiovascular outcomes in patients with AF in the new exerciser group.

**eTable 9.** Hazard ratios with 95% confidence intervals for ischemic stroke, heart failure, and all-cause death of exercise maintainer group (Yes to Yes) according to the energy expenditure (MET-min/wk).

| Exercise dose | Number | Events | IR  (1000PY) | Unadjusted HR  (95% CI) | Model 1 HR  (95% CI) | Model 2 HR  (95% CI) |
| --- | --- | --- | --- | --- | --- | --- |
| **Ischemic stroke** |  |  |  |  |  |  |
| Persistent non-exerciser | 20354 | 707 | 10.36 | 1 (Ref.) | 1 (Ref.) | 1 (Ref.) |
| < 500 MET-min/wk | 4491 | 107 | 6.73 | 0.65 (0.53-0.80) | 0.95 (0.77-1.18) | 0.97 (0.79-1.20) |
| 500-999 MET-min/wk | 8728 | 180 | 6.08 | 0.59 (0.50-0.69) | 0.78 (0.66-0.92) | 0.79 (0.67-0.93) |
| 1000-1499 MET-min/wk | 6099 | 153 | 7.68 | 0.74 (0.62-0.88) | 0.90 (0.75-1.08) | 0.91 (0.76-1.09) |
| ≥ 1500 MET-min/wk | 3516 | 94 | 8.13 | 0.78 (0.63-0.97) | 0.86 (0.69-1.07) | 0.87 (0.70-1.08) |
|  |  |  |  | p < 0.0001 | p = 0.0545 | p = 0.0778 |
| **Heart failure** |  |  |  |  |  |  |
| Persistent non-exerciser | 20354 | 4365 | 71.86 | 1 (Ref.) | 1 (Ref.) | 1 (Ref.) |
| < 500 MET-min/wk | 4491 | 815 | 56.61 | 0.79 (0.73-0.85) | 0.97 (0.90-1.04) | 0.97 (0.90-1.05) |
| 500-999 MET-min/wk | 8728 | 1553 | 58.01 | 0.81 (0.76-0.86) | 0.94 (0.88-0.99) | 0.94 (0.88-0.99) |
| 1000-1499 MET-min/wk | 6099 | 1112 | 61.71 | 0.86 (0.80-0.92) | 0.94 (0.88-1.00) | 0.94 (0.88-1.00) |
| ≥ 1500 MET-min/wk | 3516 | 601 | 57.06 | 0.79 (0.73-0.86) | 0.81 (0.75-0.89) | 0.81 (0.74-0.88) |
|  |  |  |  | p < 0.0001 | p < 0.0001 | p < 0.0001 |
| **All-cause death** |  |  |  |  |  |  |
| Persistent non-exerciser | 20354 | 1119 | 16.04 | 1 (Ref.) | 1 (Ref.) | 1 (Ref.) |
| < 500 MET-min/wk | 4491 | 108 | 6.70 | 0.42 (0.34-0.51) | 0.68 (0.55-0.83) | 0.69 (0.57-0.85) |
| 500-999 MET-min/wk | 8728 | 224 | 7.45 | 0.47 (0.40-0.54) | 0.66 (0.57-0.77) | 0.69 (0.60-0.80) |
| 1000-1499 MET-min/wk | 6099 | 131 | 6.45 | 0.40 (0.34-0.48) | 0.51 (0.43-0.62) | 0.54 (0.45-0.65) |
| ≥ 1500 MET-min/wk | 3516 | 100 | 8.48 | 0.53 (0.43-0.65) | 0.58 (0.47-0.71) | 0.60 (0.48-0.73) |
|  |  |  |  | p < 0.0001 | p < 0.0001 | p < 0.0001 |

Abbreviation: IR, incidence rate; PY, person-years; HR, hazard ratio; CI, confidence interval; MET, metabolic equivalent of task.

Model 1 adjusted for age and sex.

Model 2 adjusted for age, sex, body mass index (BMI), hypertension, diabetes mellitus, dyslipidemia, previous myocardial infarction (MI), peripheral artery disease (PAD), chronic obstructive pulmonary disease (COPD), cancer, chronic kidney disease (CKD), CHA_2_DS_2_-VASc score, use of oral anticoagulation (OAC), use of antiplatelet agents, use of statin, smoking, heavy drinking, and low income.

*** Some spelling and grammar should be corrected, i.e. line 44, 253. Line 273-5; Hard to understand the sentence, do you mean RCT`s is not possible or ethical in this population?**

**Response:** Thank you. We meant to state that a randomized controlled study evaluating the association between exercise and cardiovascular outcomes might have an ethical issue in research design by limiting lifestyle to control exercise status. Thus, this large population-based research could be an alternative to determine the influence of exercise amongst AF patients. We have revised the sentences referring to your comment.

*Discussion (Page 18, Lines 359-365)*

Lastly, this is not a randomized controlled study, and the causality and direct mechanistic link between exercise and cardiovascular outcomes would not be answered. However, there may be an ethical issue of study design as restricting the exercise (or stopping any exercise) that would make it difficult to perform as part of a randomized controlled study. Instead, our data from this large population-based research would provide evidence for exercise on clinical outcomes as part of holistic AF management.

*** Figure 1 seems to be duplicated**

**Response:** Thank you for your comment. We combined Figure 1 (the overall study scheme) and eFigure 1 (study flow diagram) since both information were relevant.

**Fig 1**. (A) Selection of study population from the National Health Insurance Service (NHIS). (B) Categorization of study population according to the change of exercise status and overall scheme of study, including clinical outcomes.

Abbreviation: AF, atrial fibrillation; CHF, congestive heart failure.

**Reviewer #4: Hyo-Jeong et al present data obtained from the National Health Information Database from the Republic of Korea. Specifically, they assess the impact of self-reported exercise habits before an initial diagnosis of atrial fibrillation and the impact of a change in exercise habits after that diagnosis using no exercise pre and post diagnosis (persistent non-exerciser) as the baseline risk group for comparison. Outcomes assessed included stroke, CHF and all-cause mortality at follow-up. Additionally, their data allowed an evaluation of the minimum amount of energy expenditure ranges (MET-min/week) in the new exerciser group across the primary outcomes of stroke, CHF and all-cause mortality. This is an interesting study from a large data-set that does contribute to our rapidly growing knowledge set on lifestyle and atrial fibrillation. The authors appropriately note the limitations of self-reported exercise habits which is a common limitation in many such studies. Overall, the study is well written but I have a few comments that should be addressed.**

*** Line 65: "piled up" is more of a vernacular phrase and should be deleted or reworded**

*** Line 65: "direct clinical consequences are less likely offered" is vague and I am unclear as to what you intended to state with this phrase**

*** Line 66 "… limited… " is vague and unclear. There are many exercise recommendations for patients with atrial fibrillation.**

**Response:** Thank you for your comments. We revised as per your comment.

*Introduction (Page 6, Lines 99-101)*

Though the link between exercise and AF-related outcomes has been supported by substantial evidence, benefits in cardiovascular morbidities led by exercise in AF patients are less likely to be offered.

*** Lines 176-177:".., but with a trend towards increased risks…" I am not proponent of using and stating there were non-significant trends especially in the context of exercise at the end range such as your >1500 MET-min/week group. This is non-significant and should be left as such. In reviewing your eTable 2, all-cause death in this group in model 1 and model 2 still have HR <1 and better than no/minimal exercise. The curve appears to flatten saying that it trends toward increasing is a bold and potentially incorrect statement.**

*** Line 187: Again there is mention of "trend"**

*** Line 232-233: I would caution and recommend not stating "appeared to be J-shape". The curve certainly flattens and this has been shown in previous studies. However, stating a true J-curve exists is difficult given the generally lower events in the end range group with widening of the error bars.**

*** Line 235-237: "Similar mechanisms would explain … in patients with AF." See above discussion/comments.**

*** Lines 244-245: Again there is a mention of trend (see above)**

**Response:** Thank you for the comment. We revised the description of the association of energy expenditure and the risks of primary outcomes as your comment.

*Result (Page 12, Linse 232-235)*

Lower risks of ischemic stroke, HF, and all-cause death were correlated with energy expenditure increment, but the associations became attenuated in the group with exercise amount above 1500 MET-min/week (**Fig 3**).

*Discussion (Page 14, Line 256-260)*

First, initiation and maintenance of exercise after AF diagnosis was associated with a significantly lower risk for HF and all-cause death. Interestingly, exercise drop-outs were associated with a lower risk of all-cause death, as well. Second, the estimates of HRs of ischemic stroke were lower in AF patients with exercise, though the association was statistically insignificant.

*Discussion (Page 16-17, Lines 307-326)*

In the present study, we found that AF patients exercising 1000-1499 MET-min/week were associated with the lower event rates for stroke, HF, and mortality in the new exerciser group. Interestingly, the association appeared to be attenuated in the range of exercise ≥1500 MET-min/week rather than persistently showing a direct inverse correlation. The mechanisms of intensive exercise to increase the risk of AF are diverse, including changes in autonomic activation [46, 47], atrial dilatation [48, 49], fibrosis, and inflammation [50, 51]. Similar physiologic changes would explain the attenuated risk-benefit in AF patients with exercise ≥1500 MET-min/week across cardiovascular outcomes, particularly in all-cause death. However, considering the wide 95% CIs in patients with ≥1500 MET-min/week, the small patient numbers in this group might be underpowered, therefore further research would be required to delineate the optimal range of exercise levels that could derive the greatest benefit among cardiovascular outcomes in patients with AF.

Exercise has been widely documented to modify potential contributors to AF, such as obesity, hypertension, and diabetes, by improving weight, glucose and lipid control, endothelial function, and lowering resting heart rate and blood pressure [52-54]. Not only can risk factors for AF but also cardiac structure and function be modified by exercise. For example, in patients with AF, the exercise group was associated with improved left atrial and ventricular function indices [11]. These mechanisms might attribute to a noticeable lower risk in HF and all-cause mortality (and possibly a lower HR in ischemic stroke) in the new exerciser and exercise maintainer groups compared to those without exercise.

**Reviewer #5: Thanks for giving me the opportunity to review this interesting manuscript titled 'Exercise habits and the risk of stroke, heart failure, and mortality in patients with incident atrial fibrillation: a nationwide population-based cohort study'.**

**1. Severe selection bias is a big concern. From a big pool of 523174 patients, they end up to a set of 66692 patients for analysis. It is not known how different are these patients from those patients in the original pool. If the authors can provide some comparison, it will be helpful.**

**Response:** We greatly appreciate your valuable comment and concern.

To estimate the outcomes according to the change of exercise status before and after incident AF, subjects who did not receive two consecutive health examinations 2 years before and after AF diagnosis were excluded. Most of the excluded subjects in this study were unavailable to get health examinations data before and after AF diagnosis to compare exercise status.

As suggested, we compared the baseline characteristics between the final study population and those who were finally excluded from this study. Excluded subjects were further categorized into subjects with only one health exam within 2 years before and after AF diagnosis and subjects with no health exam within 2 years before and after AF diagnosis (**eTable 2 in the Supplement**).

Patients with less number of health exam (0 or 1) were older, more likely to be female, had more prevalent comorbidities, and had higher CHA_2_DS_2_-VASc score than those with two consecutive health exams. We have added the baseline characteristics of excluded patients and potential implications in the Results and Discussion sections.

**eTable 2.** Baseline characteristics of newly diagnosed atrial fibrillation patients according to the number of health examinations before and after their atrial fibrillation diagnosis.

|  | Exam 0 | Exam 1 | Exam 2 | p-value |
| --- | --- | --- | --- | --- |
| No. of participants (%) | 225915 | 230567 | 66692 |  |
| Age | 69.04 ± 18.08 | 66.99 ± 12.54 | 59.52 ± 12.38 | <.0001 |
| Sex |  |  |  | <.0001 |
| Male | 107632 (47.88) | 130002 (56.38) | 42410 (63.59) |  |
| Female | 117179 (52.12) | 100565 (43.62) | 24282 (36.41) |  |
| Diabetes mellitus | 63499 (28.11) | 56669 (24.58) | 10499 (15.74) | <.0001 |
| Hypertension | 169424 (74.99) | 177900 (77.16) | 39266 (58.88) | <.0001 |
| Dyslipidemia | 83578 (37.00) | 102474 (44.44) | 2088 (3.13) | <.0001 |
| Previous MI | 19780 (8.76) | 15998 (6.94) | 2221 (3.33) | <.0001 |
| PAD | 44391 (19.65) | 52917 (22.95) | 12053 (18.07) | <.0001 |
| COPD | 63115 (27.94) | 57812 (25.07) | 10640 (15.95) | <.0001 |
| Cancer | 25609 (11.34) | 23008 (9.98) | 4197 (6.29) | <.0001 |
| CHA_2_DS_2_-VASc score | 3.90 ± 2.12 | 3.51 ± 1.89 | 1.91 ± 1.28 | <.0001 |
| Medications |  |  |  |  |
| Warfarin | 12958 (5.74) | 18494 (8.02) | 9016 (13.52) | <.0001 |
| DOAC | 5446 (2.41) | 8782 (3.81) | 3374 (5.06) | <.0001 |
| ASP | 27829 (12.32) | 39171 (16.99) | 13263 (19.89) | <.0001 |
| P2Y_12_ inhibitor | 12963 (5.74) | 16558 (7.18) | 2834 (4.25) | <.0001 |
| Statin | 25690 (11.37) | 39137 (16.97) | 9865 (14.79) | <.0001 |
| Low income * | 70312 (31.12) | 43510 (18.87) | 10817 (16.22) | <.0001 |
| **Exclusion Criteria** |  |  |  |  |
| Valvular AF | 1574 (0.70) | 2327 (1.01) | 0 (0.00) | <.0001 |
| Age < 20 years | 4575 (2.04) | 68 (0.03) | 0 (0.00) | <.0001 |
| Previous history of stroke | 59345 (26.27) | 53949 (23.40) | 0 (0.00) | <.0001 |

| Previous history of HF | 78493 (34.74) | 82916 (35.96) | 0 (0.00) | <.0001 |
| --- | --- | --- | --- | --- |

Data are presented as means ± standard deviation or No. (Percentages).

Abbreviation: MI, myocardial infarction; PAD, peripheral artery disease; COPD, chronic obstructive pulmonary disease; DOAC, direct oral anticoagulant; AF, atrial fibrillation; HF, heart failure.

* Low income denotes income belongs to the lower 20% among the entire Korean population and supported by the Medical Aid program.

** Percentages may not total 100 because of rounding.

*Result (Page 11, Line 203-207)*

Comparison between the final study population and the excluded patients, most of whom are excluded due to unavailable health examinations data within 2 years both before and after AF diagnosis, is summarized in **eTable2 in the Supplement**. Patients who received only 0 or 1 health examinations were older, more likely to be female, had more prevalent comorbidities, and higher CHA_2_DS_2_-VASc score than those with 2 health examinations.

*Discussion (Page 18, Lines 356-359)*

Seventh, the inclusion of patients only who had two consecutive health examinations before and after AF diagnosis would cause some selection bias. Our results would have limited evidence in those who could not receive health screening due to underlying diseases; thus it should be interpreted and applied cautiously to the general AF population.

**2. The commonest cause of death in patients with AF are non-cardiac death, heart failure, and stroke. In this paper, exercise status is not significantly associated with ischemic stroke, and marginally significant associated with heart failure, but very significantly associated with all-cause death. Please explain whether this is due to unadjusted confounders, which means the changes of exercise status is due to other co-morbidities, or frailty of the patients.**

**Response:** We greatly appreciate your valuable comment and concern.

First, we agree that unadjusted confounders such as comorbidities or frailty, which might indigenously contribute to exercise status changes, could affect the risk in outcomes. We, therefore, performed an analysis of primary outcomes by adjusting the Charlson Comorbidity Index (CCI) additionally **(eTable8 in the Supplement)**. The risks of ischemic stroke, heart failure, and all-cause death were consistent with the original outcome.

Second, we analyzed the causes of death to scrutinize the risk-benefit of all-cause death. In our previous study demonstrating mortality and causes of death in Korean AF patients [14], diseases of the circulatory system explain 38.0% of death. In addition, cerebral infarction (I63), acute myocardial infarction (I21), sequelae of cerebrovascular disease (I69), and heart failure (I50) were the four major specific disease entities for the cause of death belong to diseases of the circulatory system (I-code in ICD-10 system) [14]. Based on this previous study, we defined the death from major cardiovascular events like the death from I63, I21, I69, and I50. The risk of death from major cardiovascular events was significantly lower in the exercise maintainer (adjusted HR 0.71, 95% CI 0.51-0.98) and also lower in the new exerciser (adjusted HR 0.78, 95% CI 0.54-1.11), but with statistical insignificance which might be due to low event number (**eTable7 in the Supplement**). These results were largely consistent with the trends of the risk for all-cause death.

Although the risk of incident stroke was not significantly lower in the new exerciser goup and the risk of incident HF was only moderately lower in the new exerciser group compared to persistent non-exercisers, there is a possibility that the difference exists in the severity or fatality of stroke and HF between new exerciser and persistent non-exerciser which might attribute to lower risk of all-cause death.

Moreover, recent research reported that stroke-related mortality in AF patients was only 6.5-7% despite the high risk of stroke associated with AF (Eur Heart J. 2013;34(14):1061-7, Eur Heart J Qual Care Clin Outcomes. 2019;5(1):35-42, Am J Med. 2016;129(12):1278-87). Thus, it could be plausible that AF patients with exercise show a considerably lower risk in all-cause death, whereas they show an insignificant association with stroke.

Beyond these explanations, however, we agree that the substantial benefit of exercise on all-cause death might also include benefit on non-CV death, and it could be interpreted as a signal of residual confoundings such as frailty or other comorbidities of patients. Nonetheless, we cautiously appraised our results as reasonable and explainable results. We now revised the Methods, Results, and Discussion sections following your comments.

**eTable 8**. Hazard ratios with 95% confidence intervals for ischemic stroke, heart failure, and all-cause death according to the change of exercise status additionally modified by Charlson Comorbidity Index.

|  | Number | Events | IR  (1000PY) | Unadjusted HR  (95% CI) | Model 1 HR  (95% CI) | Model 2 HR  (95% CI) | Model 3 HR  (95% CI) |
| --- | --- | --- | --- | --- | --- | --- | --- |
| **Ischemic stroke** |  |  |  |  |  |  |  |
| Persistent non-exerciser | 20354 | 707 | 10.36 | 1 (Ref.) | 1 (Ref.) | 1 (Ref.) | 1 (Ref.) |
| New exerciser | 11874 | 325 | 8.06 | 0.78 (0.68-0.89) | 0.90 (0.79-1.03) | 0.92 (0.80-1.05) | 0.92 (0.80-1.05) |
| Exercise drop-outs | 11630 | 339 | 8.58 | 0.83 (0.73-0.94) | 0.90 (0.79-1.02) | 0.91 (0.79-1.03) | 0.91 (0.80-1.03) |
| Exercise maintainer | 22834 | 534 | 6.94 | 0.67 (0.60-0.75) | 0.88 (0.78-0.99) | 0.89 (0.79-1.00) | 0.89 (0.79-1.00) |
|  |  |  |  | p < 0.0001 | p = 0.1266 | p = 0.2026 | p = 0.2156 |
| **Heart failure** |  |  |  |  |  |  |  |
| Persistent non-exerciser | 20354 | 4365 | 71.86 | 1 (Ref.) | 1 (Ref.) | 1 (Ref.) | 1 (Ref.) |
| New exerciser | 11874 | 2246 | 61.71 | 0.86 (0.82-0.91) | 0.93 (0.88-0.98) | 0.94 (0.89-0.99) | 0.94 (0.89-0.99) |
| Exercise drop-outs | 11630 | 2420 | 68.51 | 0.95 (0.91-1.00) | 0.99 (0.94-1.04) | 1.00 (0.95-1.05) | 1.00 (0.95-1.05) |
| Exercise maintainer | 22834 | 4081 | 58.53 | 0.82 (0.78-0.85) | 0.93 (0.89-0.97) | 0.93 (0.89-0.98) | 0.94 (0.90-0.98) |
|  |  |  |  | p < 0.0001 | p = 0.0015 | p = 0.0167 | p = 0.0074 |
| **All-cause death** |  |  |  |  |  |  |  |
| Persistent non-exerciser | 20354 | 1119 | 16.04 | 1 (Ref.) | 1 (Ref.) | 1 (Ref.) | 1 (Ref.) |
| New exerciser | 11874 | 456 | 11.11 | 0.69 (0.62-0.77) | 0.82 (0.74-0.92) | 0.84 (0.75-0.94) | 0.83 (0.75-0.93) |
| Exercise drop-outs | 11630 | 489 | 12.13 | 0.76 (0.68-0.84) | 0.81 (0.73-0.90) | 0.83 (0.75-0.93) | 0.84 (0.75-0.93) |
| Exercise maintainer | 22834 | 563 | 7.19 | 0.45 (0.41-0.50) | 0.60 (0.54-0.67) | 0.63 (0.57-0.70) | 0.65 (0.58-0.72) |
|  |  |  |  | p < 0.0001 | p < 0.0001 | p < 0.0001 | p < 0.0001 |

Abbreviation: IR, incidence rate; PY, person-years; HR, hazard ratio; CI, confidence interval.

Model 1 adjusted for age and sex.

Model 2 adjusted for age, sex, body mass index (BMI), hypertension, diabetes mellitus, dyslipidemia, previous myocardial infarction (MI), peripheral artery disease (PAD), chronic obstructive pulmonary disease (COPD), cancer, chronic kidney disease (CKD), CHA_2_DS_2_-VASc score, use of oral anticoagulation (OAC), use of antiplatelet agents, use of statin, smoking, heavy drinking, and low income.

Model 3 adjusted for age, sex, body mass index (BMI), hypertension, diabetes mellitus, dyslipidemia, previous myocardial infarction (MI), peripheral artery disease (PAD), chronic obstructive pulmonary disease (COPD), cancer, chronic kidney disease (CKD), CHA_2_DS_2_-VASc score, use of oral anticoagulation (OAC), use of antiplatelet agents, use of statin, smoking, heavy drinking, low income, and Charlson Comorbidity Index (CCI).

**eTable 7.** Hazard ratios with 95% confidence intervals for major cardiovascular death according to the change of exercise status.

|  | Number | Events | IR  (1000PY) | Unadjusted HR  (95% CI) | Model 1 HR  (95% CI) | Model 2 HR  (95% CI) |
| --- | --- | --- | --- | --- | --- | --- |
| **Major cardiovascular death *** | |  |  |  |  |  |
| Persistent non-exerciser | 20354 | 120 | 1.72 | 1 (Ref.) | 1 (Ref.) | 1 (Ref.) |
| New exerciser | 11874 | 41 | 1.00 | 0.58 (0.41-0.83) | 0.75 (0.52-1.07) | 0.78 (0.54-1.11) |
| Exercise drop-outs | 11630 | 42 | 1.04 | 0.61 (0.43-0.86) | 0.69 (0.48-0.98) | 0.71 (0.50-1.01) |
| Exercise maintainer | 22834 | 57 | 0.73 | 0.42 (0.31-0.58) | 0.67 (0.49-0.93) | 0.71 (0.51-0.98) |
|  |  |  |  | p < 0.0001 | p = 0.0443 | p = 0.0935 |

Abbreviation: IR, incidence rate; PY, person-years; HR, hazard ratio; CI, confidence interval.

Model 1 adjusted for age and sex.

Model 2 adjusted for age, sex, body mass index (BMI), hypertension, diabetes mellitus, dyslipidemia, previous myocardial infarction (MI), peripheral artery disease (PAD), chronic obstructive pulmonary disease (COPD), cancer, chronic kidney disease (CKD), CHA2DS2-VASc score, use of oral anticoagulation (OAC), use of antiplatelet agents, use of statin, smoking, heavy drinking, and low income.

* Major cardiovascular deaths are defined as deaths due to cerebral infarction (I63), acute myocardial infarction (I21), sequelae of cerebrovascular disease (I69), and heart failure (I50).

*Method (Page 10, Lines 185-191)*

In addition, we analyzed the causes of death to scrutinize the positive risk-benefit of all-cause death using the ICD-10 code claimed at death. The most frequent four major specific disease entities, which belong to diseases of the circulatory system (I-code in ICD-10 system) and explain the cause of death among Korean AF patients, were used to define major cardiovascular death: cerebral infarction (I63), acute myocardial infarction (I21), sequelae of cerebrovascular disease (I69), and heart failure (I50) [14]. Second, we further adjusted primary outcomes by comorbidities or frailty using the Charlson Comorbidity Index (CCI) along with Model 2.

*Result (Page 13, Lines 242-253)*

Since the effect size of lowered estimates of adjusted HRs in all-cause death was larger than that of ischemic stroke and HF, we investigated whether the lower risk of all-cause death is indigenously contributed to the unadjusted confounders such as comorbidities or frailty rather comes from possible beneficial effects of exercise. First, the risk of death from major cardiovascular events was lower in the exercise maintainer (adjusted HR 0.71, 95% CI 0.51-0.98) and also lower in the new exerciser (adjusted HR 0.78, 95% CI 0.54-1.11), but with statistical insignificance which might be due to low event number (**eTable7 in the Supplement**). However, the association was consistent with the trend of the positive risk benefit in all-cause death. Second, the risks of primary outcomes according to the change of exercise habits additionally modified by CCI are described in **eTable8 in the Supplement**. The risks of ischemic stroke, heart failure, and all-cause death were consistent with the main outcome (Model 2).

*Discussion (Page 14, Lines 276-279)*

Moreover, the positive benefit of exercise on all-cause death was proven to be robust even after we additionally adjusted the association by CCI and evaluate the major cardiovascular death using the most frequent four major CVDs explain deaths of AF patients in Korea.

*Added Reference*

14.	Lee E, Choi EK, Han KD, Lee H, Choe WS, Lee SR, et al. Mortality and causes of death in patients with atrial fibrillation: A nationwide population-based study. PLoS One. 2018;13(12):e0209687. Epub 2018/12/27. doi: 10.1371/journal.pone.0209687. PubMed PMID: 30586468; PubMed Central PMCID: PMCPMC6306259.

**3. The intensity and frequency of exercise is self-reported. Whether the questionnaire is validated and how accuracy is the data collected?**

**Response:** The self-reported questionnaire of the Korean health examination was adopted and modified from the International physical activity questionnaire (IPAQ), which was developed by the World Health Organization (WHO). Both reliability and validity of this short form survey were reported in previous studies [18-21]. We revised “method-exercise evaluation” by supplementing information on survey development.

*Method (Page 7-8, Lines 128-132)*

The survey structure used for exercise evaluation was adopted and modified from the International Physical Activity Questionnaire (IPAQ), which was developed by the World Health Organization (WHO); both reliability and validity of this short form survey were reported in previous studies [18-21].

*Added Reference*

18.	Chun MY. Validity and reliability of Korean version of international physical activity questionnaire short form in the elderly. Korean journal of family medicine. 2012;33(3):144.

19.	Oh JY, Yang YJ, Kim BS, Kang JH. Validity and Reliability of Korean Version of International Physical Activity Questionnaire (IPAQ) Short Form. Journal of the Korean Academy of Family Medicine. 2007;28(7):532-41.

20.	Tomioka K, Iwamoto J, Saeki K, Okamoto N. Reliability and validity of the International Physical Activity Questionnaire (IPAQ) in elderly adults: the Fujiwara-kyo Study. J Epidemiol. 2011;21(6):459-65. Epub 2011/09/29. doi: 10.2188/jea.je20110003. PubMed PMID: 21946625; PubMed Central PMCID: PMCPMC3899462.

21.	Cleland C, Ferguson S, Ellis G, Hunter RF. Validity of the International Physical Activity Questionnaire (IPAQ) for assessing moderate-to-vigorous physical activity and sedentary behaviour of older adults in the United Kingdom. BMC Med Res Methodol. 2018;18(1):176. Epub 2018/12/24. doi: 10.1186/s12874-018-0642-3. PubMed PMID: 30577770; PubMed Central PMCID: PMCPMC6303992.

**4. All the definition of confounders and outcomes are not clearly defined. It would be good to clarify them.**

**Response:** Thank you for the comment. We revised **eTable 1 in the Supplement,** which describes detailed definitions of confounders and outcomes following your comment.

**5. Figure 1 and figure 1 should be combined, as the information of efigure 1 is also important.**

**6. efigure 1, the flow chart, all the excluded patients and reason for exclusion should be listed. I guess 523174-19148 patients did not get consecutive health examination?**

**Response:** Thank you for the comment. We combined **Figure 1** and **eFigure 1**. Also, we had clarified the excluded patients and updated the flow diagram of the study population.

**Fig 1.** (A) Selection of study population from the National Health Insurance Service (NHIS). (B) Categorization of study population according to the change of exercise status and overall scheme of study, including clinical outcomes.

Abbreviation: AF, atrial fibrillation; CHF, congestive heart failure.
