# Supplementary material for: Association between exercise habits and stroke, heart failure, and mortality in Korean patients with incident atrial fibrillation: A nationwide population-based cohort study
Source: PLoS Med. 2021 Jun 8;18(6):e1003659. doi: 10.1371/journal.pmed.1003659 (PMC8219164; doi:10.1371/journal.pmed.1003659)
Supplement: Attachment — Submitted filename: [Exercise_CVD_AF] Response to review_0321.docx [file pmed.1003659.s014.docx]

**Responses to comments:**

**• Please mention country in title and abstract.**

**Response**: Thank you for the comment. We now revised the title and abstract as per your suggestion.

*Title (Page 1, Line 1-2)*

Exercise habits and the risk of stroke, heart failure, and mortality in patients with incident atrial fibrillation: a Korean nationwide population-based cohort study

*Abstract (Page 2, Line 31-32)*

This is a nationwide population-based cohort study using data from the Korea National Health Insurance Service.

**• PLOS defines the “minimal data set” to consist of the data set used to reach the conclusions drawn in the manuscript with related metadata and methods, and any additional data required to replicate the reported study findings in their entirety. Authors do not need to submit their entire data set, or the raw data collected during an investigation. Please submit the following data: 1) The values behind the means, standard deviations and other measures reported; 2) The values used to build graphs; and 3) The points extracted from images for analysis.**

**Response**: Thank you for the comment. We retrieved our raw data from the National Health Information Database (NHID), which is only accessible online at the designated analysis centers, and applied strict regulations regarding the release. Instead, we provided all the values (i.e., event numbers, incidence rates, and hazard ratios of cardiovascular outcomes) used to reach our conclusion in main tables and figures together with supplementary materials. For example, the incidence rates and hazard ratios presented in **Fig 2**. are summarized in **Table 2**. Also, the values used to build **Fig 3**. are described in **eTable5 in the supplement**.

**• Please bullet and trim the author summary - preferably use the active voice in 1-2 points ("We investigated ..." etc).**

**Response**: Thank you for the comment. We have revised the author summary in a more active form.

*Author summary (Page 4-5, Line 62-90)*

**Why Was This Study Done?**

Atrial fibrillation (AF) is associated with an increased risk of stroke, heart failure, myocardial infarction, dementia, and death. As AF and its related health care burden are expected to surge, an integrated approach to the management of AF is an essential part of care.

Exercise has been established to benefit on AF-related outcomes, including symptoms, incidence, recurrence, burden, and quality of life. However, the specific influence of exercise on cardiovascular morbidities in patients with AF is unknown.

**What Did the Researchers Do and Find?**

This is a nationwide population-based cohort study using data from the Korea National Health Insurance Service. We performed a retrospective analysis of 66 692 patients with newly diagnosed AF who underwent two serial health examinations before and after their AF diagnosis from 2010 to 2016 in the Republic of Korea.

A self-reported questionnaire evaluated exercise status in each health examination. We investigated the incidences of ischemic stroke, heart failure, and all-cause death across the groups categorized according to the change of exercise status before and after AF diagnosis.

Patients who initiated or continued regular exercise after a diagnosis of AF were associated with a 6%-10% lower risk of HF and 16%-37% lower risk of mortality than persistent non-exerciser. For ischemic stroke, the estimates of hazard ratios were 8%-11% lower in patients with the exercise group, yet statistical significance was undetermined.

In addition, regular moderate exercise 170-240 min/week was associated with the lowest risk of cardiovascular outcomes in patients who initiate exercise after AF diagnosis.

**What Do These Findings Mean?**

AF and AF-related complications confer a major healthcare burden; thus, lifestyle modifications are advocated as part of holistic management in current AF management guidelines.

As a lifestyle intervention, we suggested evidence of exercise benefits and a specific volume of it (regular moderate exercise 170-240 min/week) that can maximize cardiovascular outcomes in AF patients. Promotion of exercise should be recommended to AF patients in order to reduce the future risk of adverse outcomes.

**• Abstract Methods and Findings:**
**1. Please include the study design, population (summary of demographic details for participants), and setting.**
**2. Please quantify the main results (please present both 95% CIs and p values).**
**3. Please include the important variables that are adjusted for in the analyses.**
**4. Please include the actual amounts and/or absolute risk(s) of relevant outcomes (including NNT or NNH where appropriate), not correlation coefficients**
**5. Final sentence of the "Methods and findings" subsection of abstract should begin "Study limitations include ..." or similar.**

**• In the Abstract conclusion, please mention specific implications for policy or clinical care substantiated by the results. Please revise Conclusion, first line (line 50) and instead write, “Initiation or continuing regular exercise after AF was associated with a lower risk of HF and mortality among patients.”**

**Response**: We have revised our main results and subgroup/sensitivity analyses by applying more robust statistical methods, inverse probability of treatment weighting (IPTW) using propensity scores (PS) calculated from all the baseline covariates across the four study groups. Weighted Cox proportional hazards models with IPTW computed the risks of the outcomes. The updated study outcomes, consistent with the original analysis, were described together with the recommended comments. We addressed the study design, setting, population, adjusted variables, p-values, and NNTs. We clarified study limitations in the last sentence of the **Methods and Findings**, and included clinical implications in the **Conclusion** as below.

*Abstract (Page 2-3, Line 26-61)*

**Background:** There is a paucity of information about cardiovascular impacts related to exercise habits after a new diagnosis of atrial fibrillation (AF). We investigated the influence of exercise habits after a new AF diagnosis on the risk of ischemic stroke, heart failure (HF), and all-cause death.

**Methods and Findings:** This is a nationwide population-based cohort study using data from the Korea National Health Insurance Service. A retrospective analysis was performed for 66 692 patients with newly diagnosed AF who underwent two serial health examinations before and after their AF diagnosis from 2010 to 2016. Individuals were divided into four categories according to performance of the regular exercise, which was investigated by a self-reported questionnaire in each health examination, before and after their AF diagnosis: persistent non-exerciser (30.5%), new exerciser (17.8%), exercise drop-outs (17.4%), and exercise maintainer (34.2%). The primary outcomes were incidence of ischemic stroke, HF, and all-cause death. Differences in baseline characteristics among groups were balanced considering demographics, comorbidities, medications, lifestyle behaviors, and income status. The risks of the outcomes were computed by weighted Cox proportional hazards models with inverse probability of treatment weighting (IPTW) during a mean follow-up of 3.4 ± 2.0 years.

New exerciser and exercise maintainer groups were associated with a lower risk of HF compared to the persistent non-exerciser group: hazard ratio (HR) 0.95, 95% confidence interval (CI) 0.90-0.99, and 0.92 (0.88-0.96), respectively (*p*<0.001). Also, patients performing exercise any time before or after AF diagnosis were associated with a lower risk of mortality compared to persistent non-exerciser; HR (95% CI) was 0.82 (0.73-0.91) for new exerciser, 0.83 (0.74-0.93) for exercise drop-outs, and 0.61 (0.55-0.67) for exercise maintainer (*p*<0.001). For ischemic stroke, the estimates of HRs were 10%-14% lower in patients with the exercise groups, yet statistically insignificant (*p*=0.057). The number needed to treat values to prevent one event of heart failure, all-cause death, and ischemic stroke was 64, 77, and 342 for new exercisers at 3 years of follow-up. Energy expenditure 1000-1499 metabolic equivalents of task-min/week (regular moderate exercise 170-240 min/week) was consistently associated with a lower risk of each outcome based on a subgroup analysis of the new exerciser group. Study limitations include recall bias introduced due to the nature of the self-reported questionnaire and restricted external generalizability to other ethnic groups.

**Conclusions:** Initiation or continuing regular exercise after AF diagnosis was associated with lower risks of HF and mortality. Regular moderate exercise 170-240 min/week was associated with the maximal benefit on cardiovascular outcomes in patients who initiate exercise after AF diagnosis. Promotion of exercise should be recommended in AF patients to lower their future risks of adverse outcomes as a part of an integrated approach to AF care.

**• In Methods and Results please update the following:**
**1. Early in methods section of main text, please state that there was no protocol or prespecified analysis plan**

**Response**: Thank you for the comment. Our study is a retrospective analysis of a nationwide population-based cohort that the number of study population retrieved from the National Health Insurance Service system. The data regarding the number, characteristics of the study population, and primary outcomes' incidences were extracted from the already existing, registered records. We utilized all the study population data relevant to the inclusion criteria rather than sampling the population. Therefore, we could not predefine the detailed study sample size and the corresponding analysis plan, thus adopted exploratory and multiple statistical analysis methods to examine our hypothesis. We now addressed that there was no predefined analysis plan and that we did our analysis in an exploratory and experimental way early in the **Methods** section.

*Methods (Page 7, Line 123-125)*

The statistical analysis plan was not pre-specified. However, the analysis was performed with a priori defined outcomes and covariates which were defined based on our previous publications before analysis [18, 19].

18.	Lee SR, Lee HJ, Choi EK, Han KD, Jung JH, Cha MJ, et al. Direct Oral Anticoagulants in Patients With Atrial Fibrillation and Liver Disease. J Am Coll Cardiol. 2019;73(25):3295-308. Epub 2019/06/30. doi: 10.1016/j.jacc.2019.04.052. PubMed PMID: 31248551.

19.	Lee SR, Choi EK, Ahn HJ, Han KD, Oh S, Lip GYH. Association between clustering of unhealthy lifestyle factors and risk of new-onset atrial fibrillation: a nationwide population-based study. Sci Rep. 2020;10(1):19224. Epub 2020/11/07. doi: 10.1038/s41598-020-75822-y. PubMed PMID: 33154443; PubMed Central PMCID: PMCPMC7645499.

**2. Please provide the actual numbers of events for the outcomes, not just summary statistics or ORs.**

**Response**: We provided the actual numbers of events for the outcomes (ischemic stroke, heart failure, and all-cause death) in main and supplementary tables according to each analysis plan. Table 2 includes actual numbers of primary outcomes according to IPTW and Cox. The actual numbers of events for the outcomes were also summarized in the supplementary tables: eTable 4 for major cardiovascular death; eTable 5 for the primary outcomes according to the exercise level; eTables 6-8 for subgroup analysis, including the event numbers of the primary outcomes according to sex, age, and CHA_2_DS_2_-VAS score; and eTables 9-10 for sensitivity analysis calculated from Cox models, respectively.

**3. Please provide p values in addition to 95% CIs**

**4. Please ensure p<0.0001 is revised to p<0.001**
**5. When a p value is given, please specify the statistical test used to determine it.**

**Response:** In the revised manuscript, we addressed the p values whenever described HRs with 95% CIs. When comparing the primary outcomes' hazard ratios according to the study groups with reference to persistent non-exercisers, *p-*values were evaluated by the likelihood ratio test. We additionally described the statistical test used to determine *p* values in the caption area of all tables.

**6. Changes in the analysis made in response to peer review comments should be identified as such in the Methods section of the paper, with rationale.**

**Response:** Thank you. We have now identified additional analysis conducted in response to peer reviews in the **Methods** section.

*Methods (Page 10, Line 193-198)*

To measure the absolute clinical benefit, we estimated absolute risk reduction (ARR) and the number needed to treat (NNT) at 3 years of follow-up to prevent one event of each cardiovascular outcome in the new exerciser (***No*** to ***Yes***), exercise drop-outs (***Yes*** to ***No***), and the exercise maintainer (***Yes*** to ***Yes***) group compared to the persistent non-exerciser (***No*** to ***No***) group. The ARR and NNT analysis were conducted by the suggestion of peer reviews.

*Methods (Page 11, Line 217-222)*

Two analyses were performed in response to the suggestion of peer reviews. We further evaluated the risk of primary outcomes by adjusting comorbidities or frailty using the Charlson Comorbidity Index (CCI) in addition to Model 2 to adjust confounding factors that might lead to a change in exercise status. The optimal amount of exercise was also demonstrated in the exercise maintainer (***Yes*** to ***Yes***) group to extend a finding of the most beneficial exercise level in the new exerciser (***No*** to ***Yes***) group.

**• The following issues have the potential to introduce bias in your study. Please address the following in your analyses or explaining how you addressed potential bias:**

**1. Reporting bias is a threat to internal validity. Please discuss the potential for systematic differences in responses between those who experienced and did not experience the outcomes of interest.**

**Response:** Thank you. We agree that a recall bias might be inevitably introduced due to the health examination survey's self-reported nature. However, for the case of reporting bias, there might be the least amount of threat since the survey was conducted before the occurrence of the primary outcomes. Indeed, the survey was not performed after the experience of the cardiovascular outcomes, but the individuals who already reported their exercise status were included and had been followed up for the evaluation of outcome occurrence. Fig 1B. illustrates the order between the survey and the experience of the outcomes. Therefore, we cautiously suggest that reporting bias might not threaten our internal validity. It is also highlighted that the surveys asking exercise habits had been conducted before the initiation of the observation in the figure below.

Rebuttal Figure 1. Statement of the time sequence from the survey point to an occurrence of the primary outcome. The survey asking exercise habits was conducted before developing ischemic stroke, heart failure, and all-cause death.

**2. In your statistical analyses, please use hierarchical/ multilevel models given that nationwide data is likely clustered at various regional levels. The potential clustering of data (e.g., among patients from the same locality or hospital) would result in spurious effect estimates and standard errors.**

**Response:** Thank you. First, our nationwide claimed data are a comprehensive information set that was collected anonymously without any sampling process. There might exist an imbalance in the regional proportion of individuals included in the study population. However, the lists of examinations, including anthropometric measurements, list of laboratory values, investigation of comorbidities, and the form of the self-reported questionnaire, were uniform across the various regions since the health examination is under supervision and management of National Health Insurance Service. Ministry of Health and Welfare designates specific hospitals that enable regular health checkups composed of the same kinds of examinations to reduce standard errors. Moreover, both reliability and validity of this survey were reported in previous studies [20-23], and we have published several studies using this nationwide cohort data without a threat of a potential cluster of data source, which might interrupt the standardization (J Am Coll Cardiol. 2019;73(25):3295-3308, Cardiovasc Diabetol. 2021;20(1):20, Stroke. 2021;52(2):511-520, etc).

20. Chun MY. Validity and reliability of Korean version of international physical activity questionnaire short form in the elderly. Korean journal of family medicine. 2012;33(3):144.

21. Oh JY, Yang YJ, Kim BS, Kang JH. Validity and Reliability of Korean Version of International Physical Activity Questionnaire (IPAQ) Short Form. Journal of the Korean Academy of Family Medicine. 2007;28(7):532-41.

22. Tomioka K, Iwamoto J, Saeki K, Okamoto N. Reliability and validity of the International Physical Activity Questionnaire (IPAQ) in elderly adults: the Fujiwara-kyo Study. J Epidemiol. 2011;21(6):459-65. Epub 2011/09/29. doi: 10.2188/jea.je20110003. PubMed PMID: 21946625; PubMed Central PMCID: PMCPMC3899462.

23.	Cleland C, Ferguson S, Ellis G, Hunter RF. Validity of the International Physical Activity Questionnaire (IPAQ) for assessing moderate-to-vigorous physical activity and sedentary behaviour of older adults in the United Kingdom. BMC Med Res Methodol. 2018;18(1):176. Epub 2018/12/24. doi: 10.1186/s12874-018-0642-3. PubMed PMID: 30577770; PubMed Central PMCID: PMCPMC6303992.

**3. Please employ more robust methods to address unobserved confounding e.g., use propensity score matching or instrumental variables.**

**Response:** As requested, we performed inverse probability of treatment weighting (IPTW) using propensity scores (PS) to balance the weights across the four study groups. We computed the risks of the outcomes by weighted Cox proportional hazards models with IPTW. In the revised manuscript, we presented the results using the IPTW method as the main results and moved the multivariable-adjusted Cox analysis results to a sensitivity analysis. Updated statistical methods and results are described in the **Methods**/**Results/Discussion** section and summarized in revised Tables and Figures. Only descriptions in the **Methods**/**Results/Discussion** section are shown in this rebuttal.

*Methods (Page 10, Line 184-198)*

Continuous variables are reported as means ± standard deviations, and categorical variables are reported as numbers (%). We performed inverse probability of treatment weighting (IPTW) using propensity scores (PS) derived from all the baseline covariates, including demographics, comorbidities, anthropometric measures, lifestyle behaviors, medications, and income status, to balance the weights across the four study groups. Maximum absolute standardized difference (ASD) < 0.1 (10%) was evaluated as a negligible difference in the baseline covariates between the study groups [27, 28]. After IPTW, the weighted incidence rates of ischemic stroke, HF, and all-cause death were computed by dividing each weighted event number by the total follow-up duration and presented as per 1000 person-years (PY). The risks of the outcomes were computed by weighted Cox proportional hazards models with IPTW. To measure the absolute clinical benefit, we estimated absolute risk reduction (ARR) and the number needed to treat (NNT) at 3 years of follow-up to prevent one event of each cardiovascular outcome in the new exerciser (***No*** to ***Yes***), exercise drop-outs (***Yes*** to ***No***), and the exercise maintainer (***Yes*** to ***Yes***) group compared to the persistent non-exerciser (***No*** to ***No***) group. The ARR and NNT analysis were conducted by the suggestion of peer reviews.

27.	Austin PC. Balance diagnostics for comparing the distribution of baseline covariates between treatment groups in propensity-score matched samples. Stat Med. 2009;28(25):3083-107. Epub 2009/09/17. doi: 10.1002/sim.3697. PubMed PMID: 19757444; PubMed Central PMCID: PMCPMC3472075.

28.	Austin PC. The use of propensity score methods with survival or time-to-event outcomes: reporting measures of effect similar to those used in randomized experiments. Stat Med. 2014;33(7):1242-58. Epub 2013/10/15. doi: 10.1002/sim.5984. PubMed PMID: 24122911; PubMed Central PMCID: PMCPMC4285179.

*Methods (Page 11, Line 211-222)*

We performed subgroup analyses of the primary outcomes according to sex, age (<65, 65 to <75, and ≥75 years), and CHA_2_DS_2_-VAS score (<3 and ≥3). P-for-interaction was estimated to assess the consistent trends in the main results among subgroups.

To verify our results' robustness, we performed sensitivity analyses by multivariable Cox proportional hazard models. Model 1 was adjusted for age and sex, and Model 2 was adjusted for body measures, comorbidities, CHA_2_DS_2_-VASc score, medications, lifestyle behaviors, and income level in addition to Model 1. Two analyses were performed in response to the suggestion of peer reviews. We further evaluated the risk of primary outcomes by adjusting comorbidities or frailty using the Charlson Comorbidity Index (CCI) in addition to Model 2 to adjust confounding factors that might lead to a change in exercise status. The optimal amount of exercise was also demonstrated in the exercise maintainer (***Yes*** to ***Yes***) group to extend a finding of the most beneficial exercise level in the new exerciser (***No*** to ***Yes***) group.

*Results (Page 12, Line 234-240)*

The baseline characteristics of each group are shown in **Table 1**. New exerciser and exercise maintainer groups tend to be younger, male-predominant, less affected with comorbidities (especially PAD, COPD, and CKD), and have lower CHA_2_DS_2_-VASc scores and healthier lifestyle behaviors. All of the covariates were well balanced after IPTW (ASDs ≤ 0.1). The multiple comparisons of covariates and the degrees of balance achievement between the four study groups are presented in **eTable3 in the Supplement.**

*Results (Page 13-14, Line 247-276)*

**Table 2** describes weighted event numbers, weighted incidence rates (per 1000 PY), and weighted hazard ratios (HRs) of ischemic stroke, HF, and all-cause death at two different time points (at 3 years and the end of study follow-up) after IPTW.

Performing exercise any time before or after AF diagnosis was related to a lower risk of mortality compared to those without exercise: HR (95% CI) was 0.82 (0.73-0.91) for new exerciser; 0.83 (0.74-0.93) for exercise drop-outs; and 0.61 (0.55-0.67) for exercise maintainer (all, *p*<0.001). The NNT values to prevent one all-cause death were 77 and 149 for exercise maintainer and new exerciser, respectively.

Initiating and maintaining exercise after AF diagnosis was associated with a lower risk of HF compared to the persistent non-exerciser group: HR (95% CI) was 0.95 (0.90-0.99) for the new exerciser group and 0.92 (0.88-0.96) for the exercise maintainer group (all, *p*<0.001). However, the exercise drop-outs exhibited a similar risk of HF with the persistent non-exerciser group. The NNT values to prevent one heart failure was 64 and 89 for exercise maintainer and new exerciser, respectively.

For ischemic stroke, participating in exercise was associated with 10-14% lower risk compared to the persistent non-exerciser group, but the precision in the estimates of HRs was statistically insignificant: HR (95% CI) 0.92 (0.80-1.05) for the new exerciser group, and 0.89 (0.79-1.00) for exercise maintainer group (*p*=0.057). According to the change of exercise status, the weighted incidence rates and risks of cardiovascular outcomes are illustrated in **Fig 2**.

Since the effect size of lowered estimates of adjusted HRs in all-cause death was larger than that of ischemic stroke and HF, we investigated whether the lower risk of all-cause death is indigenously contributed to the unadjusted confounders such as comorbidities or frailty rather than possible beneficial effects of exercise. The risk of death from major cardiovascular events was lower in the exercise maintainer (HR 0.66, 95% CI 0.49-0.90, *p*=0.040) compared to persistent non-exerciser. Both the new exerciser (HR 0.74, 95% CI 0.52-1.06) and the exercise drop-outs (HR 0.71, 95% CI 0.49-1.02) tend to show a lower HR without statistical significance, which might be due to low event numbers (**eTable4 in the Supplement**). However, the association was consistent with the trend of the positive risk-benefit in all-cause death.

*Results (Page 14-15, Line 288-301)*

Subgroup analysis was performed by sex, age, and CHA_2_DS_2_-VASc score (**eTable6-8 in the Supplement).** For ischemic stroke, there were no significant statistical interactions for sex, age, and CHA_2_DS_2_-VASc score. The incidence of HF showed an interaction with age, potentially due to an augmented lower risk of HF in the exercise maintainer group amongst those aged over 75 years (*p*-for-interaction = 0.019). The risk of all-cause death presented an interaction with sex that more benefits of exercise in male patients might explain (*p*-for-interaction = 0.048).

Sensitivity analysis validating the risks of primary outcomes according to the change of exercise habits estimated by multivariable-adjusted Cox proportional hazard models are described in **eTable9 in the Supplement**. The risks of ischemic stroke, heart failure, and all-cause death adjusted by Model 2 and additional CCI were consistent with the main analysis. Indeed, we presented **eFigure1 in the Supplement** to show various adjustment models and corresponding HRs of consistent outcomes irrespective of the variables incorporated in the adjustments. The HRs were only different in subtle between the models.

*Discussion (Page 16-17, Line 332-337)*

Moreover, the positive benefit of exercise on all-cause death was robust even after evaluating the association by another statistical method and adjusting the model, including as many covariates as possible. Also, the risk-benefit of major cardiovascular death using the most frequent four major CVDs explain deaths of AF patients in Korea [13, 14, 33-35] presented consistent trend with that of all-cause death, thereby lowered risk of all-cause death might come from positive effects of exercise.

**4. Please clearly describe how you selected adjustment variables in model 2; most of the included variables are potentially correlated. Directed acyclic graphs are instrumental in making models as parsimonious as possible.**

**Response:** We selected adjustment variables in model 2 that could be potentially associated with the development of ischemic stroke, heart failure, and all-cause death. The detailed definitions of covariates are summarized in **eTable1 in the Supplement**. There might exist collinearity among the selected adjustment variables. We, therefore, chose to calculate PS and IPTW to balance the differences across the study groups and evaluate the risks of the primary outcomes. Also, the multivariable-adjusted Cox proportional hazard model was performed as a sensitivity analysis. Although we could not present directed acyclic graphs, we presented **eFigure1 in the Supplement** to show various adjustment models and corresponding HRs of consistent outcomes irrespective of the variables incorporated in the adjustments. The HRs were only different in subtle between the models. Of note, we have published several studies analyzing the respective primary outcomes by adjusting the same variables (Hypertension. 2021;77(3):919-928, Heart Rhythm. 2020;17(3):365-371, Int J Cardiol. 2019 Oct 1;292:106-111, etc.).

*Results (Page 15, Line 299-301)*

Indeed, we presented **eFigure1 in the Supplement** to show various adjustment models and corresponding HRs of consistent outcomes irrespective of the variables incorporated in the adjustments. The HRs were only different in subtle between the models.

eFigure1 in the Supplement. Hazard ratios with 95% confidence intervals for ischemic stroke, heart failure, and all-cause death according to the change of exercise status calculated from the various multivariable-adjusted Cox proportional hazard models.

Abbreviation: CI, confidence interval.

Model 1 adjusted for age and sex.

Model 2 adjusted body mass index (BMI), smoking, heavy drinking, and low income in addition to Model 1.

Model 3 adjusted diabetes mellitus, hypertension, dyslipidemia, and previous myocardial infarction (MI) in addition to Model 2.

Model 4 adjusted peripheral artery disease (PAD), chronic obstructive pulmonary disease (COPD), cancer, and chronic kidney disease (CKD) in addition to Model 3.

Model 5 adjusted CHA_2_DS_2_-VASc score in addition to Model 4.

Model 6 adjusted the use of oral anticoagulation (OAC), use of antiplatelet agents in addition to Model 5.

*P* values were evaluated by the likelihood ratio test. The dots denote hazard ratios, and the whiskers denote 95% confidence intervals computed by multivariable Cox proportional hazards models.

**5. Please state whether you did post-hoc corrections for multiple comparisons in your analyses**

**Response:** The weighted risks of the outcomes were compared with reference to the persistent non-exerciser group. We performed multiple comparisons for the baseline characteristics of the four study groups and presented the results of the post-hoc analysis as ASDs in **eTable 3 in the Supplement**.

*Results (Page 12, Line 237-240)*

All of the covariates were well balanced after IPTW (ASDs ≤ 0.1). The multiple comparisons of covariates and the degrees of balance achievement between the four study groups are presented in **eTable3 in the Supplement.**

**eTable 3**. The multiple comparisons of the baseline characteristics between the study groups presented as absolute standardized differences.

|  | ASD | | | | | | | | | | | | | |
| --- | --- | --- | --- | --- | --- | --- | --- | --- | --- | --- | --- | --- | --- | --- |
|  | Pre IPTW | | | | | | | Post IPTW | | | | | | |
|  | A* vs B* | A vs C* | A vs D* | B vs C | B vs D | C vs D | Maximum | A vs B | A vs C | A vs D | B vs C | B vs D | C vs D | Maximum |
| **Age** | 0.216 | 0.126 | 0.412 | 0.089 | 0.192 | 0.283 | 0.412 | 0.012 | 0.020 | 0.000 | 0.009 | 0.012 | 0.021 | 0.021 |
| **Sex** | 0.183 | 0.186 | 0.498 | 0.003 | 0.308 | 0.305 | 0.498 | 0.004 | 0.005 | 0.002 | 0.001 | 0.002 | 0.003 | 0.005 |
| Hypertension | 0.049 | 0.026 | 0.066 | 0.023 | 0.017 | 0.040 | 0.066 | 0.010 | 0.010 | 0.000 | 0.000 | 0.010 | 0.010 | 0.010 |
| Diabetes mellitus | 0.046 | 0.017 | 0.083 | 0.028 | 0.038 | 0.066 | 0.083 | 0.002 | 0.000 | 0.002 | 0.002 | 0.004 | 0.002 | 0.004 |
| Dyslipidemia | 0.014 | 0.001 | 0.021 | 0.014 | 0.007 | 0.022 | 0.022 | 0.002 | 0.001 | 0.002 | 0.001 | 0.000 | 0.001 | 0.002 |
| Previous MI | 0.027 | 0.018 | 0.030 | 0.009 | 0.003 | 0.012 | 0.030 | 0.002 | 0.001 | 0.001 | 0.001 | 0.003 | 0.002 | 0.003 |
| PAD | 0.055 | 0.019 | 0.143 | 0.036 | 0.088 | 0.123 | 0.143 | 0.005 | 0.003 | 0.001 | 0.002 | 0.006 | 0.005 | 0.006 |
| COPD | 0.065 | 0.062 | 0.164 | 0.004 | 0.098 | 0.102 | 0.164 | 0.001 | 0.002 | 0.001 | 0.001 | 0.000 | 0.002 | 0.002 |
| Cancer | 0.034 | 0.011 | 0.012 | 0.023 | 0.046 | 0.023 | 0.046 | 0.002 | 0.004 | 0.000 | 0.002 | 0.002 | 0.004 | 0.004 |
| CKD (GFR ≤ 60) | 0.099 | 0.065 | 0.137 | 0.034 | 0.038 | 0.072 | 0.137 | 0.006 | 0.005 | 0.001 | 0.001 | 0.007 | 0.006 | 0.007 |
| **CHA_2_DS_2_-VASc score** | 0.251 | 0.178 | 0.531 | 0.072 | 0.281 | 0.352 | 0.531 | 0.015 | 0.012 | 0.001 | 0.002 | 0.016 | 0.014 | 0.016 |
| **OAC** | 0.005 | 0.020 | 0.005 | 0.015 | 0.000 | 0.015 | 0.020 | 0.006 | 0.003 | 0.000 | 0.003 | 0.007 | 0.003 | 0.007 |
| Warfarin | 0.028 | 0.026 | 0.034 | 0.002 | 0.007 | 0.009 | 0.034 | 0.000 | 0.001 | 0.002 | 0.003 | 0.006 | 0.003 | 0.006 |
| NOAC | 0.039 | 0.004 | 0.055 | 0.035 | 0.016 | 0.051 | 0.055 | 0.003 | 0.003 | 0.003 | 0.000 | 0.006 | 0.006 | 0.006 |
| Aspirin | 0.044 | 0.014 | 0.029 | 0.030 | 0.016 | 0.014 | 0.044 | 0.007 | 0.005 | 0.000 | 0.002 | 0.008 | 0.005 | 0.008 |
| P2Y_12_ inhibitor | 0.028 | 0.020 | 0.039 | 0.008 | 0.011 | 0.019 | 0.039 | 0.000 | 0.001 | 0.004 | 0.001 | 0.005 | 0.004 | 0.005 |
| Statin | 0.038 | 0.033 | 0.056 | 0.004 | 0.018 | 0.022 | 0.056 | 0.002 | 0.001 | 0.001 | 0.001 | 0.001 | 0.000 | 0.002 |
| BMI (kg/m^2^) | 0.041 | 0.039 | 0.076 | 0.002 | 0.036 | 0.037 | 0.076 | 0.005 | 0.004 | 0.005 | 0.001 | 0.010 | 0.010 | 0.010 |
| Waist circumference (cm) | 0.013 | 0.046 | 0.063 | 0.033 | 0.051 | 0.017 | 0.063 | 0.030 | 0.010 | 0.076 | 0.020 | 0.047 | 0.067 | 0.076 |
| Systolic BP (mmHg) | 0.051 | 0.042 | 0.086 | 0.009 | 0.034 | 0.043 | 0.086 | 0.017 | 0.000 | 0.006 | 0.017 | 0.011 | 0.006 | 0.017 |
| Diastolic BP (mmHg) | 0.009 | 0.002 | 0.020 | 0.007 | 0.029 | 0.022 | 0.029 | 0.005 | 0.002 | 0.007 | 0.002 | 0.003 | 0.005 | 0.007 |
| Fasting glucose (mg/dL) | 0.026 | 0.001 | 0.029 | 0.026 | 0.002 | 0.029 | 0.029 | 0.003 | 0.003 | 0.003 | 0.006 | 0.000 | 0.006 | 0.006 |
| Total cholesterol (mg/dL) | 0.005 | 0.001 | 0.000 | 0.006 | 0.005 | 0.001 | 0.006 | 0.001 | 0.002 | 0.002 | 0.003 | 0.001 | 0.004 | 0.004 |
| eGFR (ml/min/1.73m^2^) | 0.050 | 0.022 | 0.034 | 0.027 | 0.018 | 0.011 | 0.050 | 0.027 | 0.024 | 0.068 | 0.002 | 0.038 | 0.041 | 0.068 |
| **Alcohol consumption  (Heavy Drinker)** | 0.005 | 0.015 | 0.063 | 0.009 | 0.058 | 0.048 | 0.063 | 0.004 | 0.003 | 0.001 | 0.001 | 0.003 | 0.002 | 0.004 |
| **Smoking** | 0.162 | 0.103 | 0.380 | 0.059 | 0.214 | 0.274 | 0.380 | 0.002 | 0.002 | 0.001 | 0.000 | 0.001 | 0.001 | 0.002 |
| **Low income ^#^** | 0.053 | 0.042 | 0.120 | 0.011 | 0.067 | 0.078 | 0.120 | 0.001 | 0.000 | 0.003 | 0.001 | 0.004 | 0.002 | 0.004 |

Abbreviation: ASD, absolute standardized difference; MI, myocardial infarction; PAD, peripheral artery disease; COPD, chronic obstructive pulmonary disease; CKD, chronic kidney disease; OAC, oral anticoagulant; BMI, body mass index; BP, blood pressure; LDL, low-density lipoprotein; HDL, high-density lipoprotein; eGFR, estimated glomerular filtration rate.

* Group A denotes persistent non-exerciser, B for new exerciser, C for exercise drop-outs, and D for exercise maintainer.

\# Low income denotes income belongs to lower 20% among the entire Korean population and supported by the Medical Aid program.

**• Under the “Subgroup and additional analysis” section, 2nd paragraph (line 249), the term "trend" is used to refer to a nonsignificant P value. The term trend should be used only when the test for trend has been conducted. Please revise accordingly.**

**Response**: We have now revised our results regarding the risk of major cardiovascular deaths according to the four study groups calculated from IPTW and Cox as follows.

*Results (Page 14, Line 273-276)*

However, a lower risk of major cardiovascular death in exercise maintainer and lower estimates of HRs in the new exerciser and the exercise drop-outs were consistent with the positive risk-benefits in all-cause death (*p*=0.040).

**• Please replace "subject" with participant, patient, individual, or person.**
**• Discussion: Line 294: Please change "statistically insignificant" to "not statistically significant" or similar.**

**• Discussion: Line 355: Please change "western populations" to "populations of high-income countries" or similar**.

**Response**: We have revised the sentences as per your suggestions.

*Methods (Page 7, Line 126-127)*

We selected 523 174 patients newly diagnosed as AF between January 1, 2010 and December 31, 2016 and excluded patients with valvular AF and age under 20 years.

*Discussion (Page 17, Line 351-352)*

On the other hand, we found that the association between exercise and the risk of stroke was not statistically significant.

*Discussion (Page 19, Line 389-390)*

Of note, we categorized the study population by the change of exercise status after diagnosis of AF

*Discussion (Page 20, Line 415-417)*

Sixth, the study was conducted in an Asian population; thereby, physiological and cultural differences in exercise behavior or biological response to exercise might limit external generalizability to other ethnic groups.

**• Table 2, eTable 4,5,6,7,8, and 9: Please provide p values in addition to the 95% CIs reported.**

**Response**: We indicated all the p-values in each table.

**• Please indicate in the figure caption the meaning of the bars and whiskers in Figure 2 & 3**

**Response**: We indicated the meaning of the bars and whiskers in the caption of Fig 2 and Fig 3.

*Figure legends (Page 33, Line 682-684, 691-693)*

The bars denote weighted incidence rates, the dots denote hazard ratios, and the whiskers denote 95% confidence intervals computed by weighted Cox proportional hazards models with IPTW.

**• In the Reference section, please delete the following statement from reference 42 (line) and paste it under the appropriate COI section:**
[**www.icmje.org/coi_disclosure.pdf and declare: no support from any organisation for the submitted work; no financial relationships with any organisations that might have an interest in the submitted work in the previous three years; no other relationships or activities that could appear to have influenced the submitted work.**](http://www.icmje.org/coi_disclosure.pdf%20and%20declare:%20no%20support%20from%20any%20organisation%20for%20the%20submitted%20work;%20no%20financial%20relationships%20with%20any%20organisations%20that%20might%20have%20an%20interest%20in%20the%20submitted%20work%20in%20the%20previous%20three%20years;%20no%20other%20relationships%20or%20activities%20that%20could%20appear%20to%20have%20influenced%20the%20submitted%20work.)

**Response**: We corrected the above statement.
