# Supplementary material for: Association between exercise habits and stroke, heart failure, and mortality in Korean patients with incident atrial fibrillation: A nationwide population-based cohort study
Source: PLoS Med. 2021 Jun 8;18(6):e1003659. doi: 10.1371/journal.pmed.1003659 (PMC8219164; doi:10.1371/journal.pmed.1003659)
Supplement: Attachment — Submitted filename: [Exercise_CVD_AF] Response to review_0429.docx [file pmed.1003659.s015.docx]

**Requests from Editors:**

1. **Please revise your title according to PLOS Medicine's style. Your title must be nondeclarative. It should begin with main concept if possible. "risk" should be used only if causality can be inferred, i.e., for an RCT. Please place the study design in the subtitle (ie, after a colon). For example, “Exercise habits, stroke, heart failure, and mortality in patients with incident atrial Fibrillation in Korea: a population-based retrospective cohort study”**

**Response**: Thank you. According to the journal's recommendation, we revised our title as “Association between exercise habits and stroke, heart failure, and mortality in Korean patients with incident atrial fibrillation: a nationwide population-based cohort study.”

*Title (Page 1, Line 1-2)*

Association between exercise habits and stroke, heart failure, and mortality in Korean patients with incident atrial fibrillation: a nationwide population-based cohort study

2. **Please trim your author summary bullet points**

**Response**: Thank you. We trimmed the author summary as follows:

*Author summary (Page 4-5, Line 58-85)*

- Why Was This Study Done?

Atrial fibrillation (AF) is associated with an increased risk of stroke, heart failure, and death. As AF and its related health care burden are expected to surge, integrated management of AF is advocated as part of holistic care.

Exercise has been established to benefit AF-related outcomes, including symptoms, incidence, recurrence, burden, and quality of life. However, there is no current data providing the association between exercise and cardiovascular morbidities in patients with

AF.

- What Did the Researchers Do and Find?

We performed a retrospective analysis of 66 692 patients with newly diagnosed AF who underwent two serial health examinations before and after their AF diagnosis from 2010 to 2016 using data from the Korea National Health Insurance Service.

Individuals self-reported exercise status by a questionnaire included in each health examination and categorized into four groups according to the change of exercise status before and after AF diagnosis. We investigated and compared the incidences of ischemic stroke, heart failure, and all-cause death across the groups.

Patients who initiated or continued regular exercise after a diagnosis of AF were associated with a 6%-10% lower risk of HF and 16%-37% lower risk of mortality than persistent non-exerciser. For ischemic stroke, the estimates of hazard ratios were 8%-11% lower in patients with the exercise group, yet statistical significance was undetermined.

- What Do These Findings Mean?

As a lifestyle intervention, we suggested the potential benefits of exercise on cardiovascular outcomes in AF patients by demonstrating the association. The promotion of exercise might reduce the future risk of adverse outcomes in patients with AF.

3. **Please remove NNT values from your results as recommended by reviewer #5. These are not applicable**

**Response**: Thank you. We removed the interpretations regarding NNT values as recommended by the review. Deleted descriptions were listed in response to reviewer #5’s comment.

4. **The Data Availability Statement (DAS) requires revision. For each data source used in your study:**

**a) If the data are freely or publicly available, note this and state the location of the data: within the paper, in Supporting Information files, or in a public repository (include the DOI or accession number).**

**b) If the data are owned by a third party but freely available upon request, please note this and state the owner of the data set and contact information for data requests (web or email address). Note that a study author cannot be the contact person for the data.**

**c) If the data are not freely available, please describe briefly the ethical, legal, or contractual restriction that prevents you from sharing it. Please also include an appropriate contact (web or email address) for inquiries (again, this cannot be a study author).**

**Response**: As we commented in the previous revision, we retrieved our raw data from the National Health Information Database (NHID), which is only accessible online at the designated analysis centers with formal payment according to the period of browsing and analyzing the data and applied strict regulations regarding the release. Instead, we provided all the values (i.e., event numbers, incidence rates, and hazard ratios of cardiovascular outcomes) used to reach our conclusion in main tables and figures together with supplementary materials. We also added the website address in the Methods section.

*Methods (Page 7, Line 110-115)*

We defined a population-based cohort from the National Health Information Database (NHID), which incorporates all data from the National Health Insurance Service and covers the Republic of Korea's entire population (hereafter denoted as Korea). National Health Information Database (NHID) is only accessible online at the designated analysis centers with formal payment according to the period of browsing and analyzing the data and applying strict regulations regarding the release (<https://nhiss.nhis.or.kr/>).

5. **In your methods, please indicate how you accounted for multiple comparisons in your analyses. Did you use the Bonferroni correction or similar?**

**Response:** Thank you. We presented maximum absolute standardized difference when we evaluated the differences of baseline characteristics among groups (eTable 3 in the Supplement). We clarified this in the revised manuscript as below.

*Results (Page 12, Line 232-237)*

We evaluated the differences and balances among multiple groups by the maximum absolute standardized differences of all baseline covariates, using a threshold of 0.1 to indicate imbalance. All of the covariates were well balanced after IPTW (ASDs ≤ 0.1). The multiple comparisons of covariates and the degrees of balance achievement between the four study groups were performed by calculating ASD and presented in eTable3 in the Supplement.

6. **Please avoid using causal language such as “risk of”, “impact of”, “effect of” etc, and use “associated with” instead, in keeping with the study design of this work.**

**Response**: Thank you. We now revised several sentences that contain the causal languages.

*Abstract (Page 2, Line 27-30)*

Background: There is a paucity of information about cardiovascular outcomes related to exercise habit change after a new diagnosis of atrial fibrillation (AF). We investigated the association between exercise habits after a new AF diagnosis and ischemic stroke, heart failure (HF), and all-cause death.

*Introduction (Page 6, Line 104-106)*

Given the paucity of information about cardiovascular outcomes related to exercise habit change after a new diagnosis of AF, we aimed to investigate the association between exercise habits after a new AF diagnosis and the risk of ischemic stroke, HF, and all-cause death.

*Methods (Page 9, Line 169-172)*

To evaluate the relationship between exercise habit change and cardiovascular outcomes, we determined incident ischemic stroke and HF using the International Classification of Diseases, Tenth Revision, Clinical Modification (ICD-10-CM) code incorporating with inpatient and outpatient records.

*Methods (Page 8, Line 151-153)*

To evaluate whether there exists the optimal exercise amount related to the best cardiovascular outcomes, we analyzed the hazards of cardiovascular morbidities according to stratified energy expenditure.

*Results (Page 14, Line 271)*

*The relationship between energy expenditure and the risk of cardiovascular outcomes*

*Discussion (Page 20, Line 411-413)*

Due to limited data about exact exercise volume from the questionnaire, the relationship between endurance exercise and cardiovascular outcomes could not be evaluated.

**Comments from Reviewers:**

**Reviewer #5: This R3 manuscript is generally well written. The authors endeavored to address the reviewer's comments and they should be commended for having done a good job. However, I still have 2 major concerns with the manuscript:**

1. **This is an observational study, which only association can be derived. NNT suggests a causal effect. The authors stated they added the NNTs as the peer reviewer required, but I strongly discourage adding such an interpretation to mislead the readers.**

**Response**: Thank you. Since this is an observational study that only demonstrates a clinical association instead of a causal effect, we agree that our study could not suggest NNT values. We left out the explanations regarding NNT values as per your suggestion. We deleted the following descriptions:

*Abstract*

The number needed to treat values to prevent one event of heart failure, all-cause death, and ischemic stroke was 64, 77, and 342 for new exercisers at 3 years of follow-up

*Methods*

To measure the absolute clinical benefit, we estimated absolute risk reduction (ARR) and the number needed to treat (NNT) at 3 years of follow-up to prevent one event of each cardiovascular outcome in the new exerciser (***No*** to ***Yes***), exercise drop-outs (***Yes*** to ***No***), and the exercise maintainer (***Yes*** to ***Yes***) group compared to the persistent non-exerciser (***No*** to ***No***) group. The ARR and NNT analysis were conducted by the suggestion of peer reviews.

*Results*

The NNT values to prevent one all-cause death were 77 and 149 for exercise maintainer and new exerciser, respectively.

The NNT values to prevent one heart failure was 64 and 89 for exercise maintainer and new exerciser, respectively.

2. **The authors concluded "Regular moderate exercise 170-240 min/week was associated with the maximal benefit on cardiovascular outcomes in patients who initiate exercise after AF diagnosis". I do not agree such an interpretation. From an eyeball impression, the confidence interval of HR for different exercise groups are largely overlapped. Interaction has not ever been tested. It is also not believable 170-240min/week moderate exercise is the best recommendation to AF patients. Moreover, as the data of exercise is self-reported and only roughly classified into different categories, it is not reasonable to evaluate these effect quantitatively.**

**Response**: Thank you. As we know, no current data provides the specific intensity and amount of exercise that could be recommended to AF patients to benefit cardiovascular outcomes. Not only showing an association between exercise habit change and the clinical outcomes (ischemic stroke, heart failure, and mortality) in AF patients who harbor proarrhythmic concerns as per exercise, we tried to suggest an appropriate amount of exercise in a ballpark figure. Although our data has several limitations in measuring and grouping the exercise volume, we cautiously conclude that the hazard ratios of clinical outcomes in AF patients according to energy expenditure still have some implications; our quantitative analysis could give a clue for the future prospective studies investigating the optimal intensity and amount of exercise in arrhythmia patients and be further incorporated into the guidelines in ABC pathway.

Moreover, WHO guidelines on physical activity and sedentary behavior published in 2020 recommended 150-300 minutes of moderate-intensity aerobic physical activity to adults aged 18-64 years with or without chronic conditions for substantial health benefits, and more than 300 minutes for additional health benefits (WHO Guidelines on Physical Activity and Sedentary Behaviour, Br J Sports Med. 2020 Dec;54(24):1451-1462). Therefore, we believe 170-240min/week moderate exercise derived quantitatively from our data is a plausible value that could be suggested to AF patients.

However, we agree that the description, 170-240 min/week is an optimal dose maximizing cardiovascular outcomes in AF patients is overstated and could mislead the readers due to an explorative stratification of exercise and the lack of statistical power. Therefore, we readdressed the finding in the way of parsimonious interpretation according to your comment. Also, we explained that the energy expenditure analysis is inevitably explorative since we roughly classified the study population and energy levels into different groups in the Method section in advance. Taken together, we deleted several descriptions regarding the analysis of exercise amount and updated the Discussion paragraph by supplementing limitations based on your comment.

*Methods (Page 8, Line 151-153)*

To evaluate whether there exists the optimal exercise amount related to the best cardiovascular outcomes, we analyzed the hazards of cardiovascular morbidities according to stratified energy expenditure.

*Methods (Page 9, Line 159-162)*

The total energy expenditure level, the summation of multiplying 2.9, 4.0, and 7.0 METs by frequency of light, moderate, and vigorous exercise together with minimum duration, was stratified into <500, 500-999, 1000-1499, and ≥1500 MET-min/week in an explorative way.

*Results (Page 15, Line 295-300)*

Another sensitivity analysis verifying whether there exists a dose-response relationship between energy expenditure and the risk of cardiovascular outcomes was performed in the exercise maintainer group (***Yes*** to ***Yes***) and described in **eTable10 in the Supplement**. The levels of energy expenditure showing the lowest estimate of HRs were all different across ischemic stroke, heart failure, and all-cause death, unlike the consistent level in the new exerciser group (***No*** to ***Yes***).

*Discussion (Page 18-19, Line 353-380)*

The amount of exercise and the risk of AF shows a J-shaped relationship; for example, endurance exercise is known to increase the risk of AF [49]. However, no current data provides the specific intensity and amount of exercise that could be recommended to AF patients to benefit cardiovascular outcomes. Not only showing an association between exercise habit change and the clinical outcomes (ischemic stroke, heart failure, and mortality) in AF patients who harbor proarrhythmic concerns as per exercise, we tried to suggest an appropriate amount of exercise in a ballpark figure. We found that AF patients exercising 1000-1499 MET-min/week were associated with the lower event rates for stroke, HF, and mortality in the new exerciser group. It corresponds to performing moderate-intensity exercise 170-240 min/week and is comparable to the amount recommended to adults aged 18-64 years with or without chronic conditions, 150-300 minutes of moderate-intensity aerobic physical activity, for substantial health benefits according to WHO guidelines on physical activity and sedentary behavior [50]. Interestingly, the association appeared to be attenuated in the range of exercise ≥1500 MET-min/week rather than persistently showing a direct inverse correlation. The mechanisms of intensive exercise to increase the risk of AF are diverse, including changes in autonomic activation [51, 52], atrial dilatation [53, 54], fibrosis, and inflammation [55, 56]. Similar physiologic changes would explain the attenuated risk-benefit in AF patients with exercise ≥1500 MET-min/week across cardiovascular outcomes, particularly in all-cause death. However, considering the wide 95% CIs in patients with ≥1500 MET-min/week, the small patient numbers in this group might be underpowered. Besides, the optimal amount of exercise in the exercise maintainer group showed inconsistent energy levels that show the lowest estimates of HRs in each cardiovascular outcome. We assume that accumulated and prolonged exercise would have influenced the clinical outcomes with different magnitude of impacts. Taken together, the interpretation of this exercise dose-response relationship should be cautiously performed due to several limitations, including explorative stratification of exercise and the lack of statistical power. Our quantitative analysis could give a clue for future prospective studies investigating the optimal intensity and amount of exercise in arrhythmia patients.

*Conclusion* (*Page 22, Line 432-435)*

Regular moderate exercise 170-240 min/week might be an optimal amount that can derive maximal cardiovascular outcomes in patients who initiate exercise after AF diagnosis, but further prospective research needs to delineate the specific energy expenditure.

Added reference

50. WHO Guidelines on Physical Activity and Sedentary Behaviour. WHO Guidelines Approved by the Guidelines Review Committee. Geneva2020.
